# Emodin mitigates rheumatoid arthritis through direct binding to TNF-α

**DOI:** 10.3389/fphar.2025.1520281

**Published:** 2025-02-26

**Authors:** Dingyan Lu, Xudong Tian, Taotao Cao, Shuaishuai Chen, Chunhua Liu, Lin Zheng, Meng Zhou, Xiaoyan Peng, Yongjun Li, Ting Liu

**Affiliations:** ^1^ State Key Laboratory of Discovery and Utilization of Functional Components in Traditional Chinese Medicine, Engineering Research Center for the Development and Application of Ethnic Medicine and TCM (Ministry of Education), Guizhou Medical University, Guiyang, China; ^2^ School of Pharmacy, Guizhou Medical University, Guiyang, China; ^3^ National Engineering Research Center of Miao’s Medicines, Guizhou Medical University, Guiyang, China; ^4^ School of Biology and Engineering (School of Modern Industry for Health and Medicine), Guizhou Medical University, Guiyang, China

**Keywords:** emodin, rheumatoid arthritis, TNF-α binding, TNF-α-TNFR1 interaction, NF-κB pathway inhibition

## Abstract

Emodin has shown certain anti-rheumatoid arthritis (RA) activity in preliminary studies. However, the precise mechanisms of emodin’s anti-RA effects, particularly its direct targets, remain unclear. This study aimed to evaluate the anti-RA activity of emodin and elucidate its potential mechanisms, with a specific focus on identifying its molecular targets. Employing a collagen-induced arthritis (CIA) rat model, along with transcriptomic analysis, thermal proteome profiling (TPP) and TNF-α-induced L929 cell model, the anti-RA activity of emodin was confirmed, identifying TNF-α as a potential target. Techniques such as drug affinity responsive target stability (DARTS), cellular thermal shift assay (CETSA), Affinity ultrafiltration-liquid chromatography/mass spectrometry (AUF-LC/MS), surface plasmon resonance (SPR) and bio-layer interferometry (BLI) validated the direct binding of emodin to TNF-α. Molecular dynamics simulation, ELISA and BLI further revealed that emodin stabilizes the asymmetric trimeric structure of TNF-α, disrupting the TNF-α-TNFR1 interaction. *In vitro* assays, including luciferase reporter gene assay and TNF-α-induced MH7A cell model, demonstrated that this disruption inhibits TNF-α-induced NF-κB activation, leading to the downregulation of inflammatory mediators such as IL-6, IL-1β, and COX2. In conclusion, emodin directly targets TNF-α, stabilizing its structure and blocking TNF-α-TNFR1 interaction, which subsequently suppresses downstream NF-κB pathway activation and contributes to its potent anti-RA properties.

## Graphical Abstract

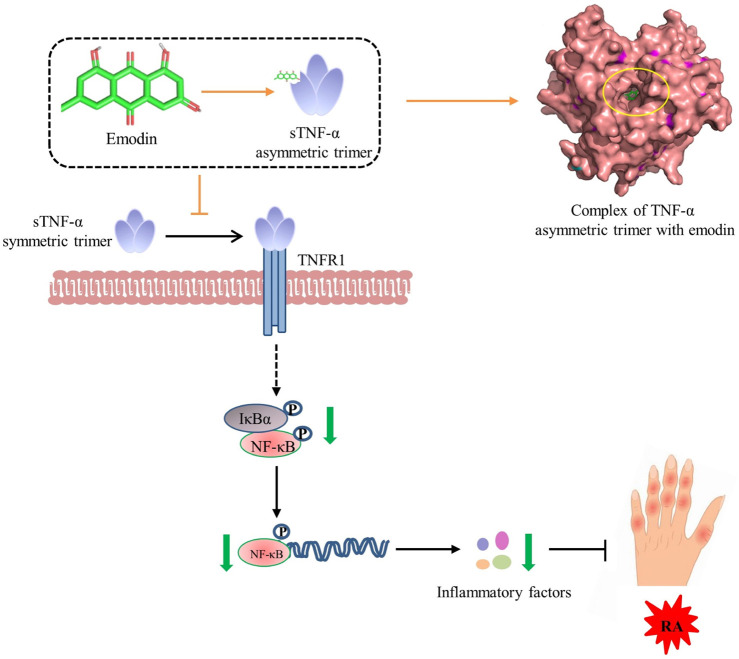

## Highlights

1. Emodin exhibits excellent anti RA activity.
2. Emodin can effectively target and inhibit the activity of TNF-α.
3. By binding directly to TNF-α and stabilizing its asymmetric trimeric form, emodin inhibits the subsequent TNF-TNFR1 signaling, and downstream NF-κB signaling pathway.
4. Emodin alleviates RA by inhibiting the NF-κB pathway via direct TNF-α binding

## 1 Introduction

Rheumatoid arthritis (RA) is characterized by chronic inflammation leading to joint damage, causing significant disability (Smolen et al., 2018). RA affects 0.5%–1% of the global population, with females experiencing a 3 to 5-fold higher prevalence than males (Smolen et al., 2016; Radu and Bungau, 2021). The exact etiology of RA is still unclear, but emerging research emphasizes the critical roles of tumor necrosis factor-alpha (TNF-α) and the nuclear factor-kappa B (NF-κB) signaling pathway in its pathogenesis (Chaabo and Kirkham, 2015; Liu et al., 2021).

In the immune-inflammatory processes occurring in the synovium and synovial fluid of RA patients, synovial macrophages release large amounts of TNF-α, IL-1β, IL-1, and IL-6 cytokines. These molecules collectively stimulate osteoclast inflammation and fibroblast-like synoviocyte (FLS) activity, promoting bone erosion (Kondo et al., 2021). Activated FLSs, in conjunction with TNF-α in the synovium, activate the NF-κB signaling pathways, inducing sustained expression of proinflammatory genes such as TNF-α, IL-6, IL-8, and IL-1β, thereby maintaining chronic inflammation and exacerbating the progression of RA (Pothacharoen et al., 2021; Xia et al., 2018). Consequently, inhibiting the activation of TNF-α and the NF-κB signaling pathway holds significant therapeutic importance in treating RA (Wysocki and Paradowska-Gorycka, 2022).

Emodin, also known as 1,3,8-trihydroxy-6-methylanthraquinone, is a natural anthraquinone compound. Notably, emodin-rich plants, including buckthorn and knotweed, have been used in traditional medicines for centuries (Evans et al., 2020). For example, *Periploca forrestii Schltr.* (*P. forrestii*), an emodin-rich species, has been widely used as dietary remedy by the “Miao” nation in china to alleviate a range of ailments, including rheumatic joint pain, soft tissue injury, and abnormal menstruation (Chen et al., 2021; Liu et al., 2018; Sun et al., 2024). Preliminary studies have demonstrated that emodin possesses a range of pharmacological activities (Semwal et al., 2021). It has been reported that emodin inhibits lipopolysaccharide-induced inflammation by suppressing the activation of NF-κB signaling pathways (Li et al., 2013; Meng et al., 2010). Further investigations suggest that emodin could reduce synovial inflammation and also alleviate symptoms of RA (Ha et al., 2011). However, despite these promising findings, comprehensive studies are required to confirm emodin’s efficacy as an anti-RA agent and to elucidate the detailed mechanisms, particularly its direct targets within the anti-RA activity.

This study aims to further explore the anti-RA potential of emodin and elucidates its underlying mechanisms. The anti-RA activity of emodin was evaluated using a collagen-induced arthritis rat model, supplemented by transcriptomic analysis and thermal proteome profiling to investigate possible mechanism and targets. To confirm the target of emodin’s anti-RA activity, techniques such as the DARTS, CETSA, AUF-LC/MS, SPR, BLI, molecular dynamics simulation and ELISA assay were employed. Additionally, a luciferase reporter gene assay and a TNF-α-induced MH7A cell model were used to verify its ability to antagonize target activity.

## 2 Material and methods

### 2.1 Chemicals and reagents

Emodin was purchased from the National Institutes for Food and Drug Contro (Beijing, China, cas no. 518-82-1, batch no. 110756, 96%). Methotrexate (MTX) was obtained from Beijing InnoChem Science & Technology Co., Ltd. (Beijing, China, cas no. 59-05-2, batch no. A82743, 99%). *P. forrestii* was purchased from the Wandongqiao Chinese herbal medicine market (Guizhou, China, harvested in 2020) and was identified as dried roots of *P. forrestii* by Associate Professor Chunhua Liu from the School of Pharmacy, Guizhou Medical University (specimen number: GY20200506). Lipopolysaccharide (LPS) and incomplete Freund’s adjuvant (IFA) were purchased from Sigma‒Aldrich (MO, United States). Type II bovine collagen was obtained from Chondrex (Washington, United States). ELISA kits for RF, TNF-α, IL-6, and IL-1β were obtained from ZCIBIO Technology Co., Ltd. (Shanghai, China). The NF-κB-RE NlucP/NF-κB-RE/Hygro] plasmid and the Nano-Glo^®^ Luciferase Assay System were purchased from Promega (Madison, WI, United States). Actinomycin D (Act D), trypsin, and trypsin inhibitor were obtained from Glpbio (Montclair, CA, United States). UCB-9260 was purchased from MCE (New Jersey, United States, cas no. 1515888-53-5, batch no. HY-133122, 98.38%). Surfactant P20 and S-series CM5 chips were obtained from Cytiva (Washington, United States). Human TNF-α recombinant protein and Human TNFR1 recombinant protein were purchased from Peprotech (NJ, United States). Anti-GAPDH antibody and horseradish peroxide-conjugated secondary antibody were obtained from Thermo Fisher Scientific (Waltham, United States). Anti-TNF-α antibody for ELISA was purchased from abcam (Cambridge, United Kingdom). Anti-TNF-ɑ antibody for Western blot was acquired from proteintech. (Wuhan, China). Antibodies against p65, p-p65, IκBα, and p-IκBα were purchased from Cell Signaling Technology (Danvers, MA, United States). Genemore G-MM-IGT Biotinylation Kit was obtained from Genemore (Jiangsu, China). Streptavidin (SA) sensor and Aminopropylsilane (APS) sensor were acquired from Sartorius (Shanghai, China).

### 2.2 Animal experiment

Both female and male animals can be used to establish CIA models for studying the anti-RA effects of drugs (Li et al., 2023; Meng et al., 2023; Liu et al., 2023). However, many studies have reported that female rodents are more susceptible to developing autoimmune complications compared to males (Smolen et al., 2016; Radu and Bungau, 2021; Babaahmadi et al., 2023). Consequently, female rats are commonly used in RA-related research to ensure a higher success rate in model establishment (Babaahmadi et al., 2023; Liu et al., 2022; Nashaat et al., 2023). Based on this consideration, female Wistar rats were selected to establish CIA models in this study. The animal and experimental protocols were conducted according to the Guide for the Care and Use of Laboratory Animals and approved by the animal care and use committee of Guizhou Medical University (China), which conforms to the guidelines from Directive 2010/63/EU of the European Parliament on the protection of animals used for scientific purposes (Approval Number 2000824). 36 female Wistar rats (SPF grade, 200–220 g) were obtained from Changsha Tianqin Biotechnology Co., Ltd. (SCXK (Xiang) 2022-0011, Changsha, China) and housed in an SPF-grade animal facility (12 h light/dark cycle, temperature: 23°C ± 2°C, relative humidity: 55% ± 5%). All animals were allowed to eat and drink freely during the experiment.

After a week of acclimation, the rats were divided into six groups of six rats each: a control group, a model group, a methotrexate group (MTX, 0.9 mg/kg), and low (20 mg/kg), medium (40 mg/kg), and high (80 mg/kg) dose groups of emodin. The dosage of emodin was determined based on previous studies (Liu et al., 2022; Zhang et al., 2021). Each rat’s tail hair was clipped, and they were subsequently injected subcutaneously at the tail base with 0.2 mg of a collagen emulsion (comprising IFA and a type II collagen solution at 1 mg/mL). A booster injection of the same emulsion was administered 7 days after the initial immunization to enhance the immune response and establish the CIA rat model. Rats in the control group received physiological saline injections similarly.

After the successful establishment of the CIA rat model, the MTX group was dosed orally with MTX twice weekly, whereas the emodin groups take orally a suspension of emodin prepared with 0.5% carboxymethyl cellulose sodium (CMC Na) every day. The control and model groups were administered a 0.5% CMC-Na solution orally. This regimen lasted for 35 days, with weekly measurements of the left foot toes’ volume. Following the final dose, the rats were fasted for 12 h (with water allowed) before collecting blood from the abdominal aorta (under pentobarbital sodium anesthesia) for ELISA determination of serum levels of rheumatoid factor (RF), TNF-α, IL-1β, and IL-6. Ankle joints were harvested for histopathological analysis.

### 2.3 Cell culture and selection of emodin dosage

RAW264.7 cells were sourced from the American Type Culture Collection (ATCC, Manassas, VA, United States). L929 mouse fibroblasts and HEK293T cells were obtained from the Cell Bank of the Chinese Academy of Sciences (Shanghai, China), while MH7A cells were purchased from Guangzhou Gini Biotechnology Co., Ltd. (Guangzhou, China). L929 cells were cultured in 1,640 medium supplemented with 10% fetal bovine serum (FBS). MH7A, HEK293T, and RAW264.7 cells were cultured in DMEM enriched with 10% FBS. All cell lines were incubated at 37°C in a humidified chamber containing 5% CO_2_ to ensure optimal growth conditions.

For the selection of emodin dosage, cells were treated with different concentrations of emodin for 24 h. Cell viability was assessed with an CCK-8 assay (Glpbio, Shanghai, China) according to the manufacturer’s instructions. The results showed that emodin did not inhibit the growth of L929 cells at concentrations below 15 μM (Supplementary Figure S4), MH7A cells at concentrations below 7 μM (Supplementary Figure S6), and HEK293T cells at concentrations below 10 μM (Supplementary Figure S7). Therefore, 5 μM, 10 μM, and 15 μM were selected for L929 cells, and 1 μM, 2.5 μM, and 5 μM were selected for MH7A cells and HEK293T cells.

### 2.4 Transcriptomic analysis

For Transcriptomic analysis, MH7A cells were seeded in 6-well plates and allowed to adhere overnight. The cells were then divided into 3 groups: a control group treated with vehicle, a model group treated with TNF-α (30 ng/mL), and an emodin group treated with emodin (5 μM) in the presence of TNF-α (30 ng/mL). Following treatment for 24 h, RNA was extracted using TRIzol reagent, and RNA sequencing was conducted by Applied Protein Technology Co., Ltd. (Shanghai, China). Differentially expressed genes (DEGs) were identified using a p-value threshold of 0.05 and an absolute log2 (fold change) greater than 1. Pathway enrichment analyses were performed with KOBAS 3.0 (http://bioinfo.org/kobas/).

### 2.5 Thermal proteome profiling

For thermal proteome profiling, MH7A cells in the logarithmic growth phase were rinsed twice with ice-cold PBS. The cells were then lysed using RIPA buffer (containing 1% PMSF) and centrifuged at 12,000 rpm for 20 min at 4°C. The cell supernatants were divided into two groups, one treated with emodin (80 μM) and the other with DMSO. These samples were incubated for 30 min at room temperature and then subjected to heating for 3 min at 72°C. Proteomic analysis was subsequently performed by Applied Protein Technology Co., Ltd. (Shanghai, China). Candidate proteins were identified based on a log2 (Fold Change) greater than 2 or less than −2, with a p-value less than 0.05, and a minimum value exceeding 2.

### 2.6 Cell viability assay

The TNF-α-induced L929 cell model is commonly used for studying the activity of TNF-α inhibitors (Sun et al., 2020). Briefly, cell viability was assessed using the CellTiter-Glo^®^ Luminescent Cell Viability Assay kit. Briefly, L929 cells (1.5 × 10^5^ cells/mL) were seeded in 96-well plates and allowed to adhere overnight. For treatment preparation, emodin was dissolved at concentrations of 0, 5, 10, and 15 μM, each subsequently mixed with Act D (1 μg/mL) and TNF-α (7.5 ng/mL). These emodin mixtures were then incubated at 37°C for 30 min before being applied to the cells. After a treatment for 12 h, the supernatant was removed, and 100 µL of CellTiter-Glo^®^ reagent was added to each well. The plate was subjected to shaking for 4 min to facilitate mixing and then allowed to stabilize at room temperature for 8 min. Chemiluminescent signals were recorded, employing a multimode microplate reader (Variskan Lux, Thermo Fisher, United States).

### 2.7 Drug affinity responsive target stability

RAW264.7 cells are widely used in drug target discovery for inflammatory arthritis, especially in analyses employing DARTS or CETSA techniques (Liu et al., 2019; Geng et al., 2024). After culturing RAW264.7 cells (1.5 × 10^5^ cells/mL) for 24 h, the cells were incubated with 1 μg/mL LPS for an additional 24 h. Subsequently, the supernatant was discarded, and cells were lysed using RIPA lysis buffer. The lysate was centrifuged at 140,00× g for 20 min at 4°C, and the supernatant was then collected for further analysis. The lysate was incubated with 80 μM emodin at room temperature for 30 min. A control group was established by incubating the lysate with DMSO. Protease was subsequently added to the lysates at different ratios (1:0, 1:100, 1:300, 1:1,000, 1:3,000, 1:10,000), followed by incubation at room temperature for 30 min. Afterward, 2 μL of 20× protease inhibitor solution was added to each sample. Finally, samples were mixed with 6× loading buffer and denatured at 100°C for 10 min. The influence of emodin on TNF-α enzyme stability was evaluated via Western blot analysis, as detailed in Section 2.15.

### 2.8 Cellular thermal shift assay

The lysate was prepared according to the procedure described in Section 2.7 and then incubated with 80 μM emodin at room temperature for 30 min. For the control, DMSO was used to treat another set of lysate samples. These resultant were subjected to incubation at varying temperatures: room temperature, 60°C, 64°C, 68°C, 72°C, 76°C, and 80°C, each for 3 min. After incubation, the samples were centrifuged at 140,00× g for 10 min at 4°C, and the supernatants were collected. These supernatants were mixed with 6× loading buffer, denatured at 100°C for 10 min, and subjected to Western blot analysis, as outlined in Section 2.15.

### 2.9 Affinity ultrafiltration-liquid chromatography/mass spectrometry

Following the protocol detailed in our previous research (Cao et al., 2022), dry *P. forrestii* (1 kg) was ground to a powder and subjected to extraction with 70% ethanol, using solvent-to-material ratios of 8, 6, and 6 (w/v), with each refluxing period lasting 1.5 h. The ethanol was evaporated after filtration, and the resulting *P. forrestii* extract underwent affinity ultrafiltration. In brief, the *P. forrestii* extract was dissolved in 75% methanol to achieve a final concentration of 100 mg/mL. Subsequently, the TNF-α group was prepared by mixing 12.5 µL of this extract, 50 µL of TNF-α solution (100 μg/mL), and 187.5 µL of Tris-HCl buffer (pH 8.7, 50 mM), and the mixture was incubated at 37°C for 30 min. For the control group, heat-treated TNF-α was used. Additionally, the *P. forrestii* extract group was prepared by combining 12.5 µL of the extract with 237.5 µL of Tris-HCl buffer (pH 8.7, 50 mM) without adding TNF-α, and this mixture was also incubated at 37°C for 30 min. The reference standard for emodin were also prepared and subjected to the same treatment. After incubation, the samples were centrifuged at 13,000 g at room temperature using Amicon Ultra 0.5 mL, 3 kDa centrifugal filters. The filtrates, after being rinsed with distilled water and a methanol-water solution, were dried under nitrogen and redissolved in methanol for mass spectrometry analysis.

Mass spectrometry analysis utilized a Vanquish UHPLC system coupled with a Q-Exactive Plus Orbitrap mass spectrometer (Thermo Fisher Scientific, Waltham, MA, United States). A Thermo Fisher Scientific Symmetry C18 column (100 mm × 2.1 mm, 1.9 µm) maintained at 25°C was used for separation. The mobile phase consisted of 0.1% (v/v) formic acid in water (A) and acetonitrile (B). The injection volume for all samples was 2 μL. The elution program was as follows: 0–30 min, 5% B; 7–18 min, 70% B; 18–19.5 min, 95% B; and 19.5–24 min, 5% B. The flow rate was set at 300 μL/min. Mass spectrometry parameters included a spray voltage: 3.5 kV (+)/2.5 kV (−), a vaporizer temperature of 350°C, and a scan range of 100–2,000. The method applied was full ms-ddms2, with MS1 resolution at 7,000 and MS/MS resolution at 15,000, using stepped normalized collision energy (NCE) of 20, 40, 60. Sheath gas was set at 35 arb, AUX gas at 10 arb, and the capillary temperature at 320°C. Data acquisition and processing were conducted using Thermo Xcalibur 4.4 (Thermo Fisher Scientific). The enrichment factor was calculated as the ratio of emodin’s peak intensity in the TNF-α group or the control group to that in the *P. forrestii* extract group.

### 2.10 Surface plasmon resonance analysis

SPR analysis was performed with a Biacore T200 system (Cytiva, Washington, United States). Initially, emodin was prepared in PBS-Surfactant P20 solution (PBS-P) with 5% DMSO, achieving concentrations ranging from 3.125 to 50 μM. Following this, recombinant hTNF-α protein was immobilized on a CM5 sensor, achieving an immobilization level of 8,700 response units. For the analysis, PBS-P with 5% DMSO were pumped over the chip at 20 μL/min. This process involved a 100-s contact time and a 150-s dissociation phase. To account for the solvent effects, calibration curves were established using PBS-P with 5% DMSO. The assessment of binding kinetics and kinetic fitting was executed using the Biacore T200 evaluation software, ensuring precise and accurate analysis of emodin’s interaction with TNF-α.

### 2.11 Bio-layer interferometry analysis

The hTNF-α recombinant protein was biotinylated as follows: 200 μg/mL of protein was mixed with 10 mM biotinylation reagent and incubated at room temperature for 60 min. The mixture was then passed through a desalination column pre-balanced with PBS, discarding the flow-through. The biotinylated protein was eluted and collected using PBS. For the BLI experiment, after pre-wetting the SA sensor, biotinylated TNF-α protein was solidified onto the sensor. Then different concentrations of emodin (0.6, 1.9, 5.6, 16.7, 50 μM) were introduced for analysis. The SA sensor was balanced, combined and dissociated in turn, and the data acquisition and analysis were performed using the Octet BLI Discovery analysis software (Octet ^®^ R4, Sartorius, Germany).

### 2.12 Molecular dynamics simulation

The protein structures of TNF-α (PDB IDs: 1TNF, 2az5, 6ooy, 6ooz, 6op0) and the molecular structure of emodin (in mol2 format) were sourced from the PDB and ZINC databases, respectively. Chem3D 19.0’s MM2 force field was used for energy minimization. Molecular docking was performed with AutoDock Vina 1.1.2, and results were visualized and analyzed using PyMol 2.3.0. GROMACS v2020.6 conducted molecular dynamics simulations on the TNF-α asymmetric trimer and its complex with emodin after initial preparation steps that included removal of ligands and water, addition of missing atoms, and neutralization of charge. Simulations ran for 100 ns, using NVT and NPT ensembles at 300 K and 1 bar, respectively. Results were processed using GROMACS tools and visualized with Qtgrace software.

### 2.13 Evaluation of the impact of emodin on TNF-α-TNFR1 interaction

ELISA was used to assess the effect of emodin on the interaction between TNFR1 and TNF-α (Sun et al., 2020; Mascret et al., 2021). Briefly, microtiter plates were coated with TNFR1 (2.5 μg/mL) in PBS overnight at 4°C. Wells were washed three times with PBS/0.05% Tween 20 (PBST) and blocked with 200 μL of PBST containing 1% BSA for 60 min at RT. After blocking, wells were washed as described above. Serial dilutions of emodin (in 0.5% DMSO) were mixed with a fixed quantity of TNF-α in PBS/BSA 1% and incubated 2 h at 37°C. The mix was then added to the wells, followed by incubation for another 2 h. After washing, wells were incubated with anti-TNF-α antibody (1:1,000) for 120 min, followed by five additional washes with PBST. A horseradish peroxide-conjugated secondary antibody was then added and incubated for 120 min. Following another set of washes, 100 μL of TMB substrate solution was added to the wells, and the reaction was quenched with 100 μL of 2 N sulfuric acid. Absorbance was measured using a fluorescence microplate reader (Thermo Fisher Scientific). Wavelength was set at 450 nm.

BLI was employed to further evaluate the impact of emodin on the interaction between TNFR1 and TNF-α (Chen et al., 2022; Lian et al., 2013). in briefly, the APS sensor was utilized to immobilize the TNFR1 protein, and the TNF-a protein solution (12.5 μg/mL) was analyzed with or without emodin (12.5 μM). The BLI analysis comprised three main steps: baseline (PBS buffer), binding (TNF-α solution with or without emodin), and dissociation (PBS buffer). The parameters of instrument and data processing are the same as those described in Section 2.11.

### 2.14 Luciferase reporter gene analysis

HEK293T cells (1.5 × 10^5^ cells/mL) were seeded in 96-well plates and cultured for 12 h. Subsequently, a 10 μL transfection mixture per well, containing FUGENE 6 transfection reagent and pNL3.2. NF-κB-RE plasmid in a 3:1 ratio, was then added to the cells and gently mixed by shaking for 30 s. After 24 h of transfection, the cells were treated for 1 h with TNF-α, which had previously been preincubated with emodin at concentrations of 1 μM, 2.5 μM, and 5 μM for 30 min. As a positive control, cells were also treated with TNF-α preincubated with 10 μM UCB-9260 for the same duration. Subsequently, the cells were then cultured for an additional 5 h. Plates were then brought to room temperature for 10 min, after which 100 μL of Nano-Glo^®^ Luciferase Assay substrate, at a 1:50 substrate-to-buffer ratio, was added to each well. After a 3 min reaction, luminescence signals were measured using a multimode microplate reader (Variskan Lux, Thermo Fisher, United States).

### 2.15 Western blot analysis

MH7A cells were seeded in 6-well plates and cultured for 24 h. TNF-α was preincubated with emodin at concentrations of 1 μM, 2.5 μM, and 5 μM for 30 min. The cells were treated with the TNF-α/emodin mixture, for an additional 24 h, followed by two PBS washes. The lysed with RIPA buffer containing 1% PMSF. Cell lysates were collected and centrifuged at 120,00× g for 20 min at 4°C. The supernatant was collected, and protein concentrations were determined using the BCA method. Protein was mixed with 6× loading buffer and boiled for 10 min. 30 μg of protein samples were separated by sodium dodecyl sulfate‒polyacrylamide gel electrophoresis (SDS‒PAGE) and transferred to a PVDF membrane. The membrane was blocked in 5% BSA solution at room temperature for 3 h, followed by incubation overnight at 4°C with primary antibodies against TNF-α (1:2,000), p65 (1:1,000), p-p65 (1:1,000), IκBα (1:1,000), p-IκBα (1:1,000), or GAPDH (1:5,000). After washing with TBST, the membrane was incubated with secondary antibodies conjugated to horseradish peroxidase for 2 h at room temperature. Following TBST washing, the membrane was visualized using a gel imaging system (Thermo Fisher Scientific). The relative protein expression levels were quantified by analyzing the grayscale ratio of the target protein to GAPDH for each sample.

### 2.16 Cell immunofluorescence

Cover slips were placed in 6-well plates with a minimal amount of culture medium. MH7A cells were seeded and cultured for 24 h. After 24 h of treatment with TNF-α that had been preincubated with emodin at concentrations of 1 μM, 2.5 μM, and 5 μM, the cells were washed three times with PBS, with each wash lasting 5 min. Cells were then fixed with 4% paraformaldehyde for 20 min at room temperature, permeabilized with 0.5% Triton X-100 for another 20 min, and washed again as before. Blocking was performed at 4°C using 5% BSA for 30 min. Overnight incubation at 4°C with a 1:50 dilution of p65 antibody followed. After washing with PBS, cells were incubated with secondary antibodies for 2 h at room temperature. DAPI was applied for nuclear counterstaining and left in the dark for 10 min. Images were captured using a Zeiss LSM 900 laser scanning confocal microscope (Carl Zeiss AG, BW, Germany).

### 2.17 Real-time quantitative polymerase chain reaction assay

MH7A cells were treated with TNF-α/emodin mixture as described in Section 2.15. Then total RNA was extracted using the Total RNA Extraction Kit (Promega, United States) and reverse transcribed into cDNA using the PrimeScript RT Kit (TaKaRa, Japan) according to the manufacturer’s instructions. The resulting cDNA products were sequentially subjected to qPCR by use of a TB Green^®^ Premix DimerEraser TM kit (Takara, Kyoto, Japan) with a CFX96 Real-Time PCR system (Bio-Rad, Hercules, United States). The cycling parameters were 95°C for 30 s, 40 cycles of 95°C for 15 s, 60°C for 15 s, and 72°C for 30 s. The primer sequences are detailed in Table 1. The relative gene expression (fold change) was calculated using the 2^−ΔΔCT^ method. Data were normalized to GAPDH levels.

**TABLE 1:** Primer sequences for target genes.

Genes	Forward	Reverse
*GAPDH*	ACA​ACT​TTG​GTA​TCG​TGG​AAG​G	GCC​ATC​ACG​CCA​CAG​TTT​C
*IL-6*	CCT​GAA​CCT​TCC​AAA​GAT​GGC	TTC​ACC​AGG​CAA​GTC​TCC​TCA
*IL-1β*	TTC​GAC​ACA​TGG​GAT​AAC​GAG​G	TTT​TTG​CTG​TGA​GTC​CCG​GAG
*COX2*	CTG​GCG​CTC​AGC​CAT​ACA​G	CGC​ACT​TAT​ACT​GGT​CAA​ATC​CC

### 2.18 Statistical analysis

Data are presented as the mean ± SD. Statistical analysis was performed using the SPSS 23.0 (Chicago, IL, United States). One-way ANOVA was employed, and results were graphed using GraphPad Prism 8.2. *p* < 0.05 was considered statistically significant.

## 3 Results

### 3.1 Emodin mitigates RA responses and symptoms and improves histopathology in a CIA rat model

**FIGURE 1:**
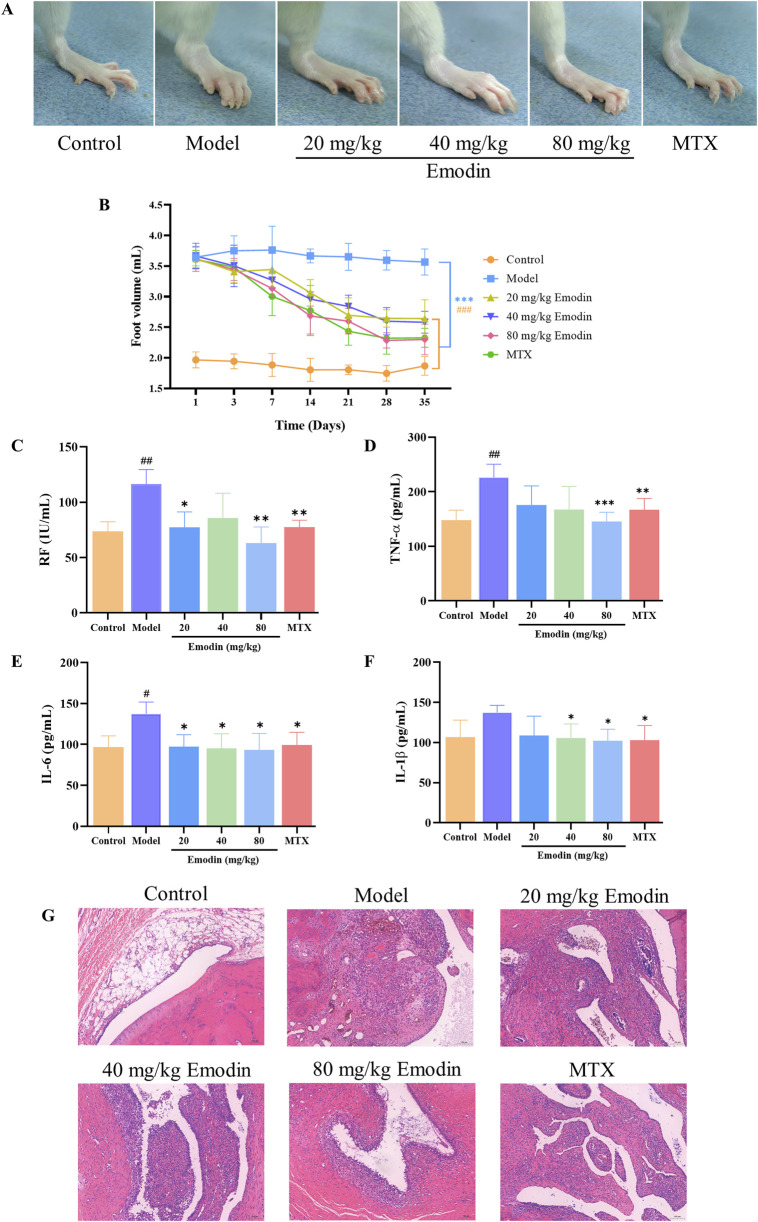
Emodin improves RA responses in CIA rats. **(A)** Swelling of the ankle joint in rats. **(B)** Volume of the toes in rats. **(C)** Levels of RF in rat serum. **(D)** Levels of TNF-α in rat serum. **(E)** Levels of IL-1β in rat serum. **(F)** Levels of IL-6 in rat serum. **(G)** Histopathological changes in the ankle joint of rats (×100 magnification). ^#^ *p* < 0.05, ^##^ *p* < 0.01, ^###^ *p* < 0.001 compared to the control group; *p < 0.05, **p < 0.01, ***p < 0.001 compared to the model group.

A CIA rat model was utilized to evaluate the anti-RA activity of emodin. As shown in Figure 1, emodin significantly alleviated swelling in the left ankle joint of CIA rats compared to the model group (Figure 1A). It also significantly reduced the volume of the toes (*p* < 0.001, Figure 1B), and decreased serum levels of rheumatoid factor (RF), TNF-α, IL-6, and IL-1β (*p* < 0.05, Figures 1C–F). Furthermore, emodin ameliorated degeneration and necrosis of synovial lining cells and lessened inflammatory cell infiltration within joint tissues (Figure 1G). These findings demonstrate that emodin effectively mitigates arthritis symptoms in the CIA rat model, underscoring its potential as a promising anti-RA agent.

### 3.2 Key pathways identified through RNA-seq analysis

**FIGURE 2:**
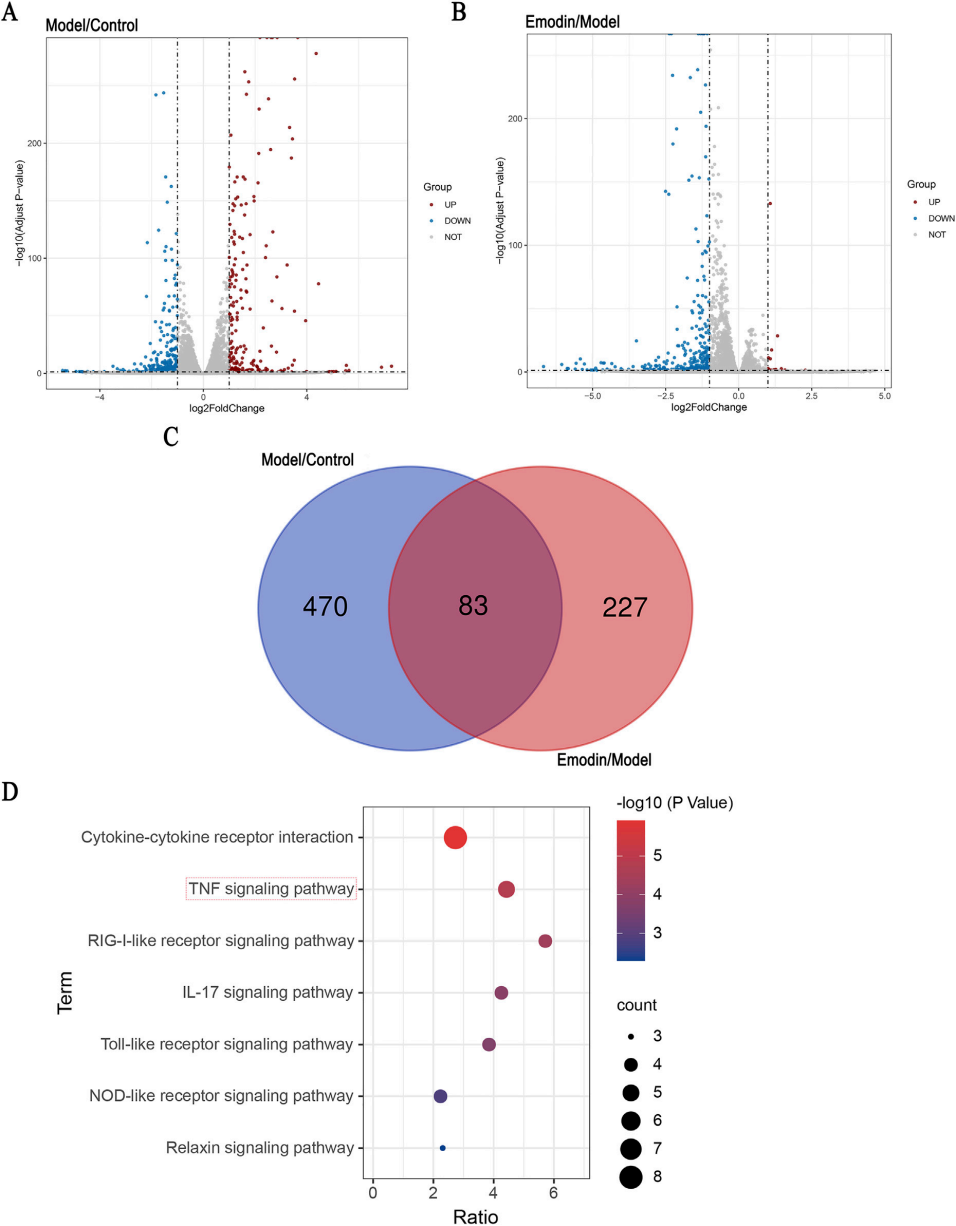
Identification of DEGs and KEGG pathway enrichment. Identification of signaling pathway of emodin. **(A)** Distribution of the log2 Fold Change and Adjusted p-values (log 10 Adjust P-value) for comparison between control and model groups. **(B)** Distribution of the log2 Fold Change and Adjusted p-values (log 10 Adjust P-value) for comparison between model and emodin groups. **(C)** The inner section of the diagram has a set of DEGs associated with emodin. **(D)** KEGG enrichment of shared DEGs.

MH7A cells are human-derived rheumatoid arthritis synovial fibroblast cells, making them more representative of human physiology compared to animal-derived cells. As a classic cell line widely used in rheumatoid arthritis RA research (Ni et al., 2023), MH7A cells are particularly well-suited for studying RA-related mechanisms. Accordingly, RNA-seq analysis was conducted using MH7A cells to explore the potential signaling pathways underlying the anti-RA effect of emodin. The results showed that, in the model group, the expression levels of 243 DEGs were significantly upregulated, whereas those of 310 DEGs were significantly downregulated. Following the intervention with emodin, the expression levels of 16 DEGs were upregulated, while 294 were downregulated (Figures 2A, B). Of these, 83 DEGs were identified across the control, model, and emodin groups (Figure 2C). KEGG pathway analysis of these 83 DEGs highlighted the TNF signaling pathway as significantly impacted (Figure 2D), suggesting its pivotal role in mediating emodin’s therapeutic effects against RA.

### 3.3 TNF-α is identified as a putative target of emodin

**FIGURE 3:**
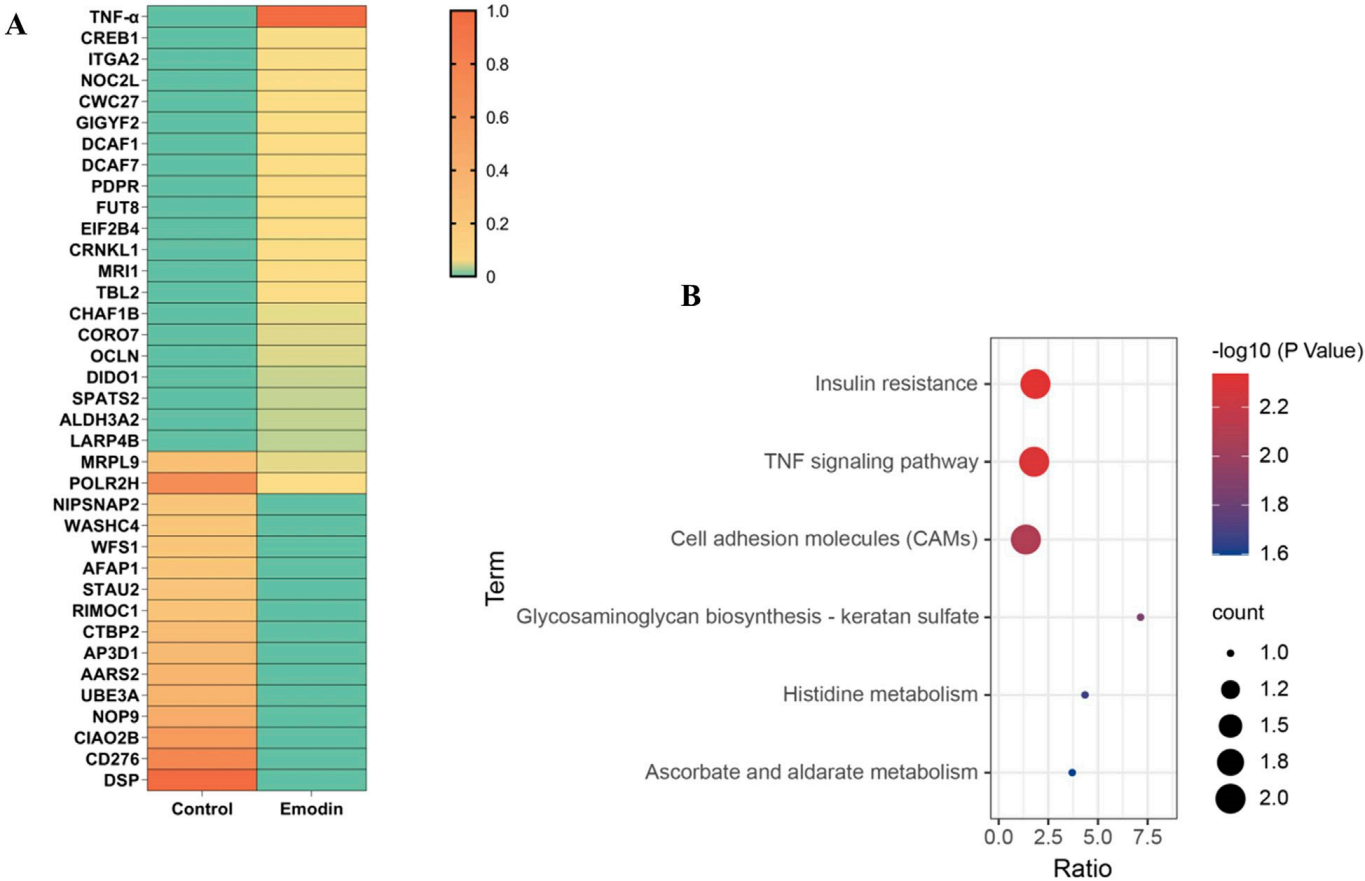
Candidate protein analysis. **(A)** Heatmap plot. **(B)** KEGG enrichment.

TPP can identify target proteins by detecting differences in thermostability caused by ligand binding, and has been widely applied to identify drug targets (Qiu et al., 2022; Savitski et al., 2014). Therefore, TPP analysis was used to investigate potential targets of emodin for anti-RA. Analysis of TPP data revealed that 37 candidate proteins showed altered abundances between the control and emodin groups (Figure 3A). Among these, TNF-α demonstrated the most significant increase in relative protein abundance following emodin treatment. Additionally, KEGG enrichment analysis identified the TNF signaling pathway as the most significantly affected pathway (Figure 3B). These findings suggest that TNF-α may be a primary target of emodin’s anti-RA action.

### 3.4 Emodin enhances TNF-α-induced L929 cell viability

**FIGURE 4:**
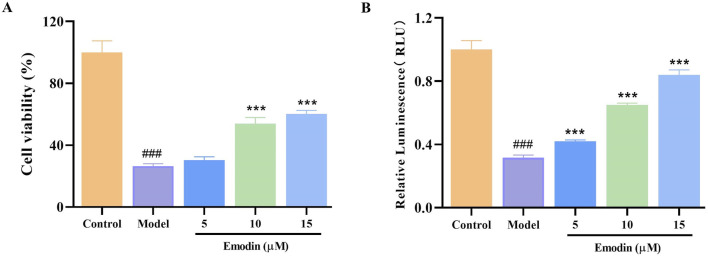
Effect of emodin on TNF-α-induced L929 cell viability. **(A)** Effect of emodin on the TNF-α-induced L929 cell viability was assessed by CCK-8 method. **(B)** Effect of emodin on TNF-α-induced L929 cell viability was assessed by CellTiter-Glo^®^ Luminescent Cell Viability Assay kit. ^###^ *p <* 0.001 compared to the control group; ***p < 0.001 compared to the model group.

L929 cells are particularly susceptible to necroptosis when stimulated by TNF-α, making them an ideal model for identifying compounds that can inhibit TNF-α function (Sun et al., 2020; Dietrich et al., 2021). Therefore, the TNF-α-induced L929 cell model was used to investigate the effect of emodin on TNF-α activity. Results indicated that emodin significantly enhanced survival and viability in TNF-α-treated L929 cells (*p* < 0.001, Figures 4A, B). These findings suggest that emodin may protect L929 cells from TNF-α-induced damage through the inhibition of TNF-α activity.

### 3.5 Emodin enhances TNF-α enzymatic stability and thermal stability

**FIGURE 5:**
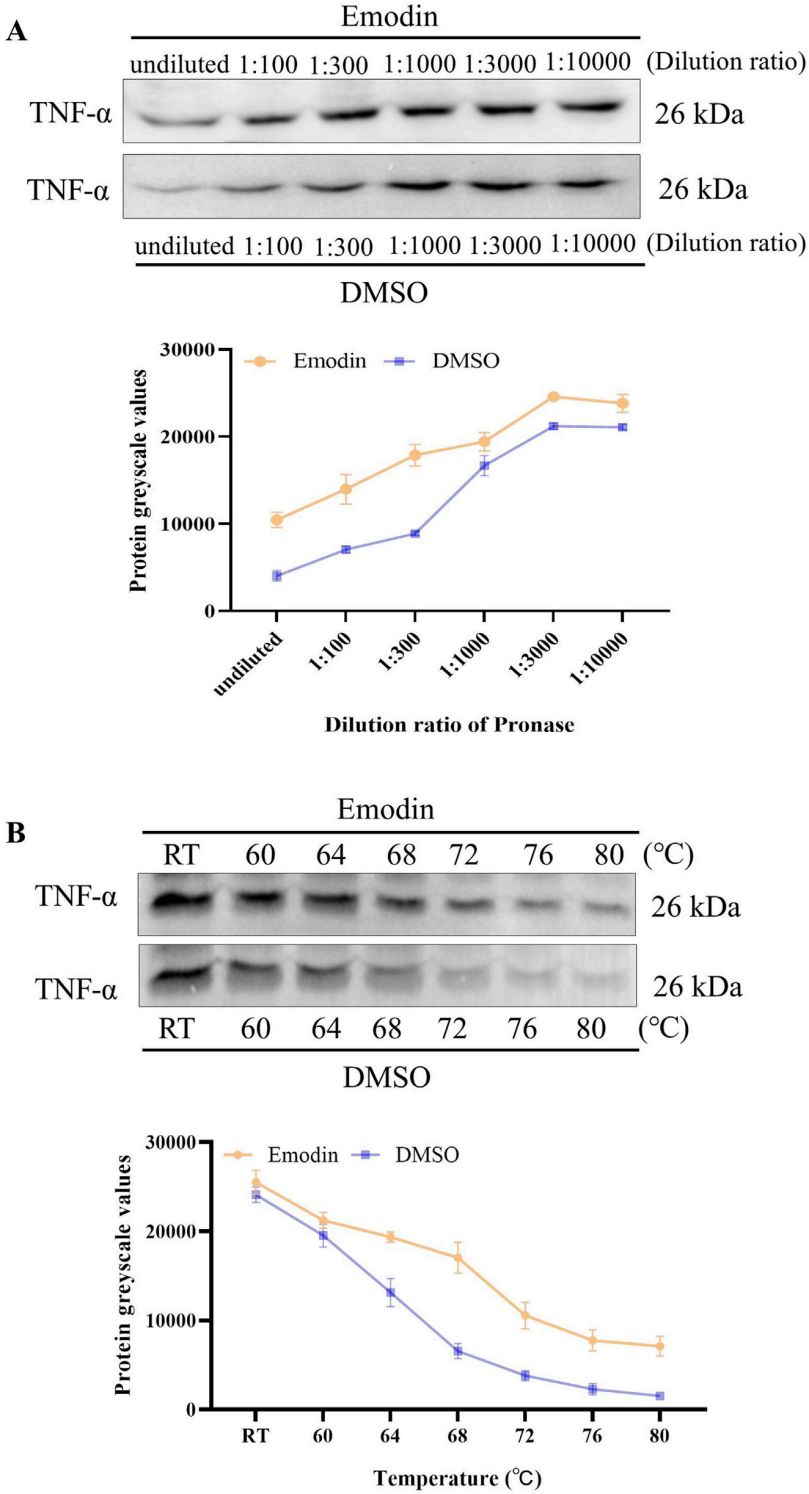
Emodin enhances TNF-α enzymatic stability and thermal stability. **(A)** DARTS detection of emodin’s impact on TNF-α enzymatic stability. **(B)** CETSA detection of emodin’s effect on TNF-α thermal stability.

DARTS and CETSA were utilized to evaluate the effects of emodin on the enzymatic and thermal stability of TNF-α. DARTS analysis revealed that emodin reduced TNF-α hydrolysis by chymotrypsin compared to the DMSO control (Figure 5A). CETSA findings indicated that emodin increased the thermal stability of TNF-α (Figure 5B). Collectively, these results suggest that emodin binds to TNF-α, enhancing its thermal stability and resistance to proteolytic degradation. Since DARTS and CETSA are only preliminary screening methods and require a large amount of protein, we selected RAW264.7 cells based on previous studies (Liu et al., 2021; Geng et al., 2024). However, RAW264.7 cells are of murine origin, and the TNF-α expressed in these cells has slight structural differences from human TNF-α (hTNF-α). To further confirm the binding of emodin to hTNF-α, AUF-LC/MS, SPR, and BLI were employed for validation.

### 3.6 Emodin strongly and specifically binds to TNF-α

**FIGURE 6:**
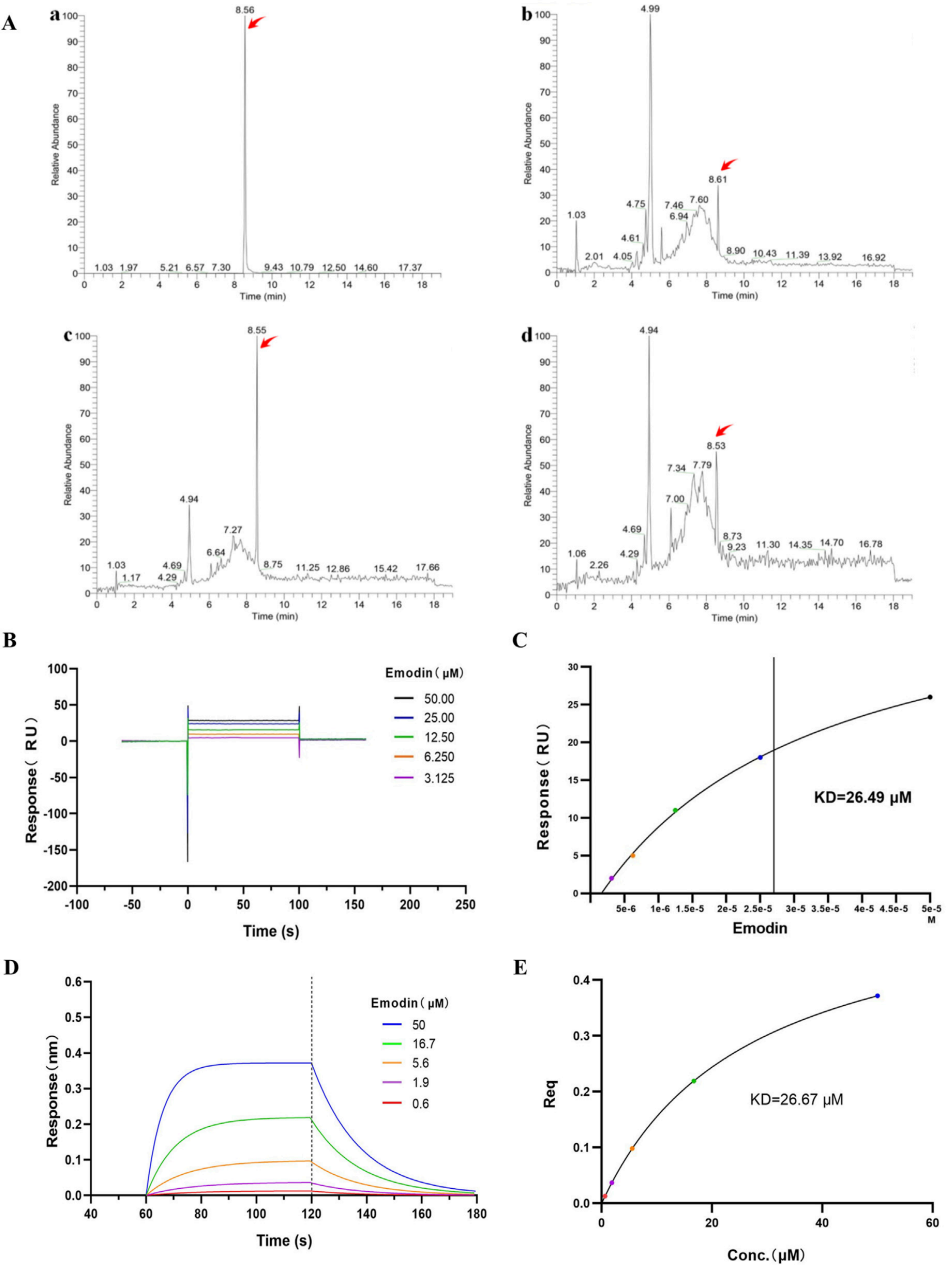
Emodin binds to TNF-α. **(A)** Affinity ultrafiltration mass spectrometry detecting the binding interaction between emodin and TNF-α. (The TIC chromatograms of standard substance (a), *P. forrestii* extract group (b), TNF-α group (c) and control group (d). Peaks indicated by red arrows represent the response peaks of emodin). **(B)** SPR detection of the binding interaction between emodin and TNF-α. **(C)** Fitting calculation of the KD value between emodin and TNF-α. **(D)** BLI detection of the binding interaction between emodin and TNF-α. **(E)** Fitting calculation of the KD value between emodin and TNF-α.

AUF-LC/MS integrates affinity capture, ultrafiltration, and LC-MS to effectively screen compounds by their interaction with target proteins, thereby indirectly indicating the binding dynamics between natural chemicals and proteins (Yang et al., 2022; Lan et al., 2021). *P. forrestii*, a traditional Chinese medicine (TCM) used in the Miao ethnic area for treating RA, contains emodin as one of its components (Liu et al., 2018; Zhang et al., 2024). Therefore, AUF-LC/MS was used to investigate whether TNF-α could be enriched into emodin from *P. forrestii*. Results from AUF-LC/MS demonstrated that emodin was detected in both the *P. forrestii* extract and the TNF-α groups, exhibiting retention times and mass spectrometric behaviors consistent with the standard substance (secondary mass spectrum detailed in Supplementary Figure S1). Notably, after affinity ultrafiltration, the enrichment factor was 5.6 times higher in the TNF-α group compared to the control group (Figure 6A; Table 2).

**TABLE 2:** Peak intensity of emodin m/z 269.05

Groups	t_R_/min	Peak intensity^a^	Enrichment factor^a^
*P. forrestii* extract group	8.61	2.00 × 10^6^	1
TNF-α group	8.55	2.61 × 10^6^	1.3
Control group	8.53	4.52 × 10^5^	0.23

^a^ Note: Peak intensity of emodin m/z 269.05. The enrichment factor was defined as of emodin’s peak intensity in the TNF-α, or control group relative to that in the *P. forrestii* extract group.

Additionally, SPR and BLI techniques were used to investigate the binding ability of emodin with TNF-α, and their affinity was calculated. SPR and BLI analysis revealed the dissociation constant (KD) of approximately 26.49 μM (Figures 6B, C) and 26.67 μM (Figures 6D, E) for emodin with TNF-α, indicating a strong and specific interaction. Collectively, these results confirm emodin’s potent interaction with TNF-α, supporting its role as a targeted inhibitor that directly binds to TNF-α to exert its anti-RA effects.

### 3.7 Binding with emodin enhances the stability of TNF-α asymmetric trimer

**FIGURE 7:**
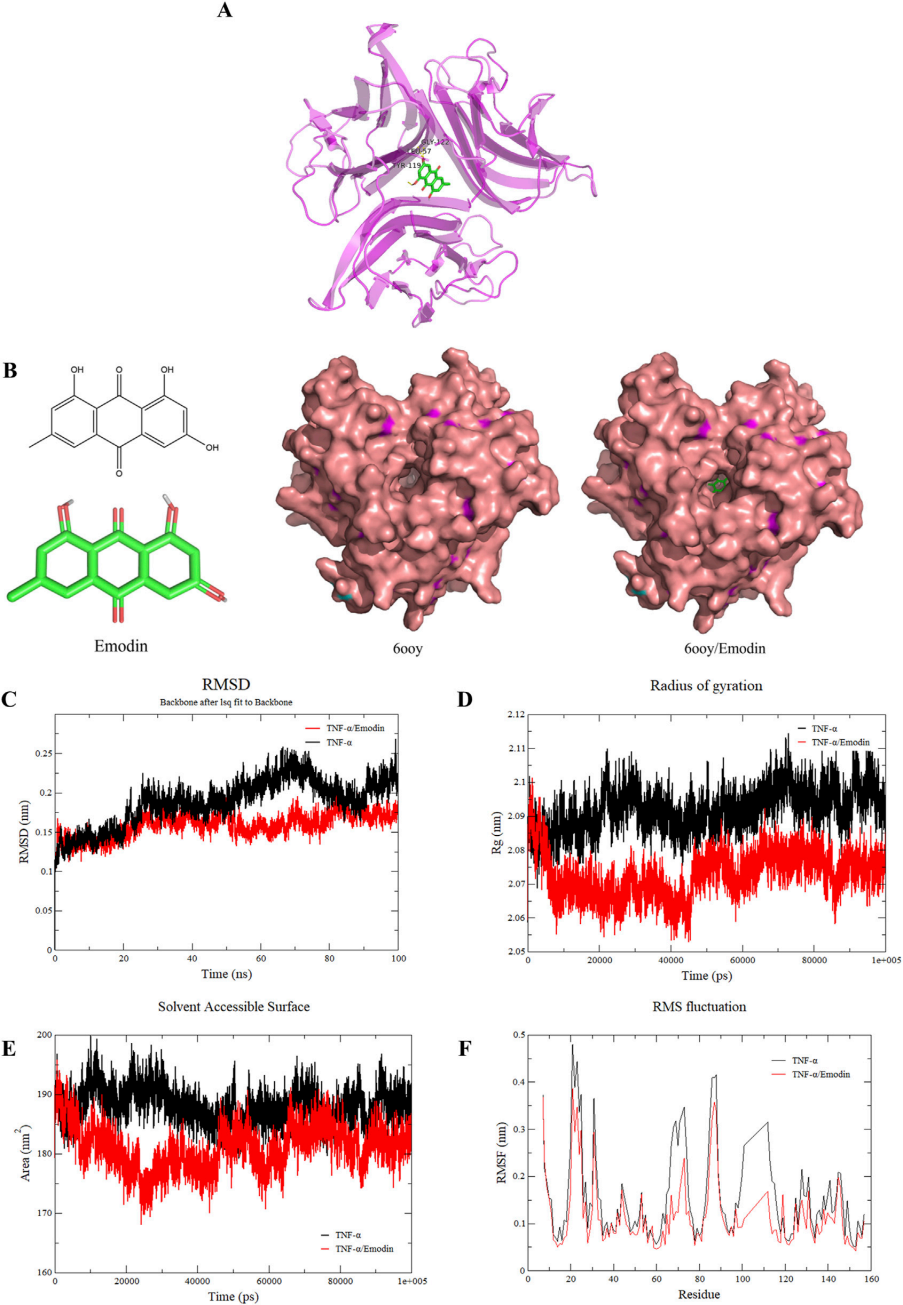
Emodin increases the stability of TNF-α asymmetric trimers. **(A)** Molecular docking results of emodin with the TNF-α asymmetric trimer (6ooy). **(B)** Structural diagram of emodin, TNF-α asymmetric trimer (6ooy), and the TNF-α/Emodin complex. **(C)** Changes in RMSD values of TNF-α and TNF-α/Emodin complex. **(D)** Changes in Rg values of TNF-α and TNF-α/Emodin complex. **(E)** Changes in SASA values of TNF-α and TNF-α/Emodin complex. **(F)** Changes in RMSF values of TNF-α and TNF-α/Emodin complex.

Dynamic simulations were conducted to unveil the effects of emodin binding on TNF-α. Through molecular docking with published TNF-α structures (PDB IDs: 1TNF, 2az5, 6ooy, 6ooz, and 6op0), it was identified that emodin exhibited the highest docking score with the TNF-α asymmetric trimer (6ooy), reaching −10.2 kcal/mol (Table 3). Furthermore, the binding of emodin to TNF-α (PDB IDs: 1TNF, 2az5, 6ooy, 6ooz, and 6op0) involves hydrogen bond interactions with the amino acids LEU-57 and TYR-119. The binding of emodin to 6ooy primarily involves hydrogen bond interactions with the amino acids LEU-57, TYR-119, and ELY-122 (Figures 7A, B). The results of molecular dynamics simulations indicated that the TNF-α/Emodin complex exhibited lower average RMSD, Rg, SASA, and RMSF compared to the TNF-α (Figures 7C–F), suggesting that emodin binding enhances the stability of the TNF-α asymmetric trimer (6ooy).

**TABLE 3:** Emodin molecular docking results with TNF-α

PDB ID	1TNF	2az5	6ooy	6ooz	6op0
Docking scores (kcal/mol)	9.2	−7.7	−10.2	−9.6	−9.9

### 3.8 Emodin blocks the interaction between TNF-α and TNFR1

**FIGURE 8:**
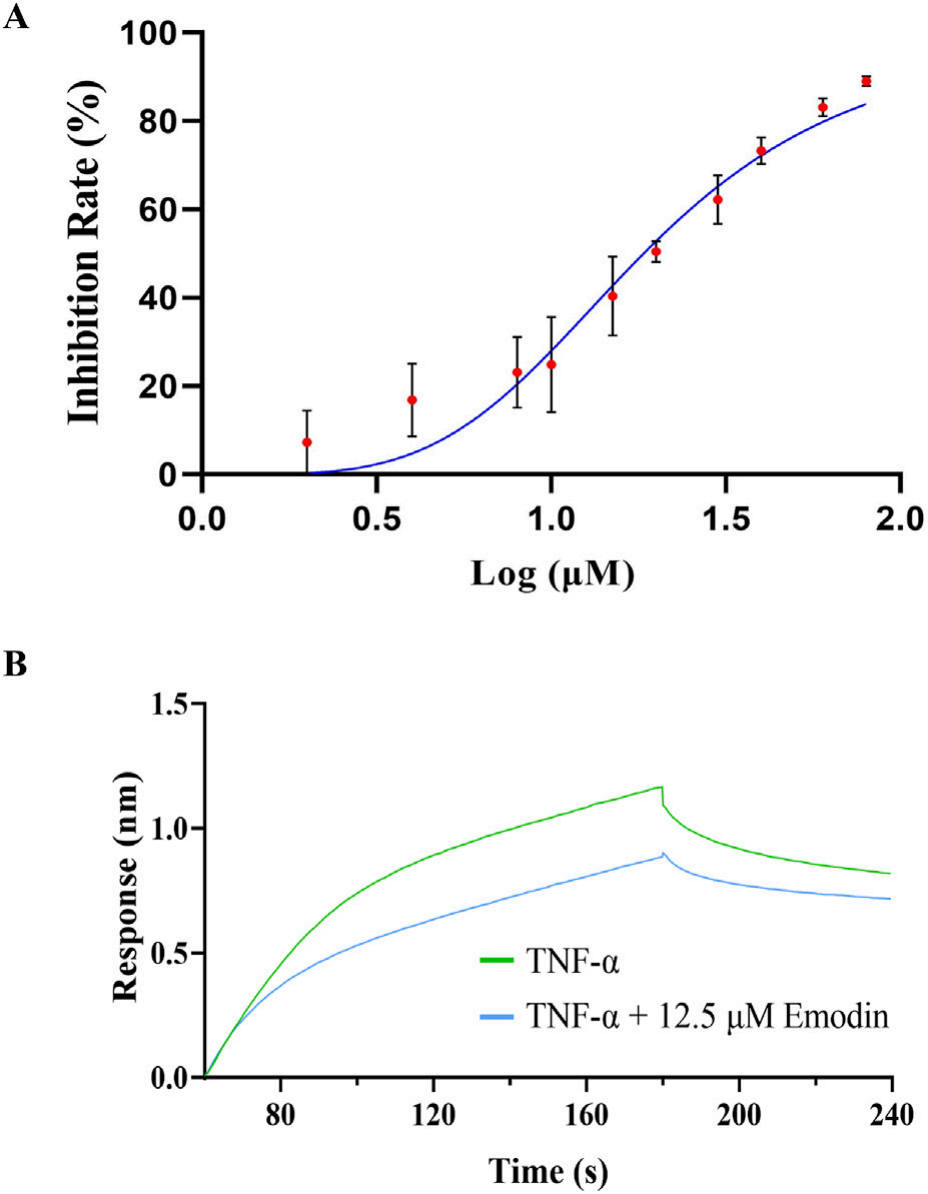
Emodin inhibits the interaction between TNFR1 and TNF-α. **(A)** Effect of emodin on the interaction between TNFR1 and TNF-α was assessed by ELISA. **(B)** Effect of emodin on the interaction between TNFR1 and TNF-α was assessed by BLI.

To further investigate whether the direct binding between emodin and TNF-α block the interaction between TNF-α and TNFR1, ELISA and BLI assay were conducted. The results indicated that emodin binds tightly to TNF-α, effectively blocking its interaction with TNFR1, with an IC_50_ of 19.14 ± 2.23 μM (Figure 8).

### 3.9 Emodin suppresses TNF-α-induced NF-κB activation

**FIGURE 9:**
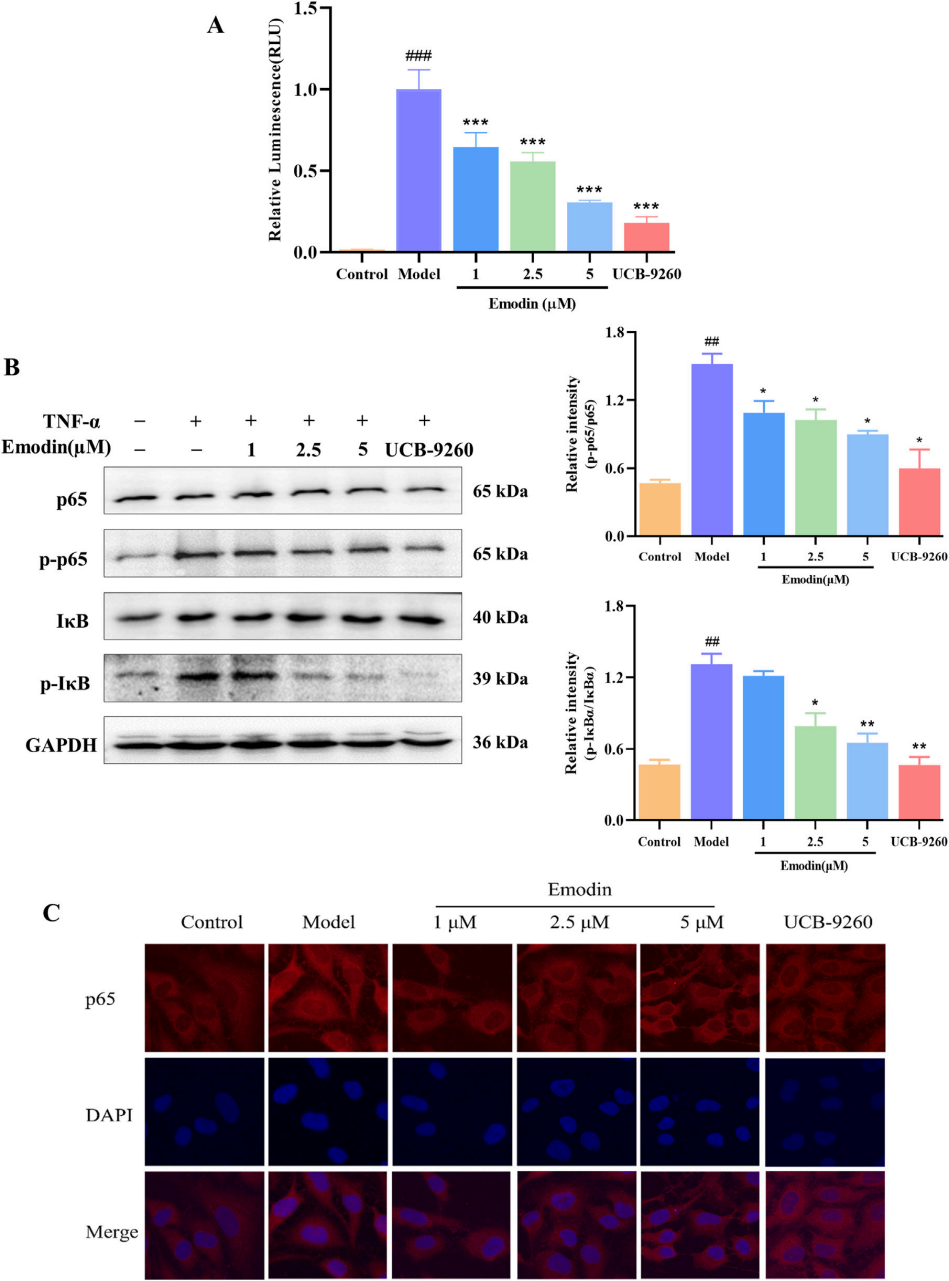
Emodin suppresses TNF-α-induced NF-κB activation. **(A)** Emodin inhibits TNF-α-induced NF-κB activation in HEK293T cells transfected with pNL3.2. NF-κB-RE plasmid. **(B)** Emodin suppresses the phosphorylation levels of p65 and IκBα in TNF-α-induced MH7A cells. **(C)** Emodin suppresses p65 nuclear translocation in TNF-α-induced MH7A cells (×400). ^##^ *p* < 0.01, ^###^ *p* < 0.001 compared to the control group; *p < 0.05, **p < 0.01, ***p < 0.001 compared to the model group.

The blockade of TNF-α-TNFR1 interaction suggests that emodin inhibits TNF-α-induced NF-κB activation. Building on these findings, we further explored the mechanism behind emodin’s anti-RA activity. First a luciferase reporter gene assay was employed to examine the effects of emodin on the NF-κB signaling pathway. Results indicated that TNF-α significantly increased chemiluminescent signals indicative of NF-κB pathway activation, which were markedly attenuated by co-incubation with emodin (*p* < 0.001, Figure 9A).

To validate these findings, we used the classic TNF-α-induced MH7A cell model known for studying anti-RA effects. Western blot analysis was performed to assess the inhibitory effects of emodin on the TNF-α-induced phosphorylation of p65 and IκBα. As expected, TNF-α significantly increased the phosphorylation of these proteins, indicating activation of the NF-κB pathway. However, in the presence of emodin, there was a significant reduction in the phosphorylation levels of both p65 and IκBα in TNF-α-stimulated MH7A cells (*p* < 0.01, Figure 9B).

Furthermore, cellular immunofluorescence was used to assess p65 nuclear translocation in TNF-α-induced MH7A cells. In cells treated with TNF-α, there was a marked increase in p65 translocation to the nucleus. Contrastingly, emodin treatment significantly reduced p65 nuclear translocation in TNF-α-stimulated MH7A cells (*p* < 0.001, Figure 9C). Taken together, these result supports the conclusion that emodin suppresses the NF-κB signaling pathway activation through its interaction with TNF-α.

### 3.10 Emodin decreases *IL-6*, *IL-1β*, and *COX2* mRNA levels in TNF-α-induced MH7A cells

**FIGURE 10:**
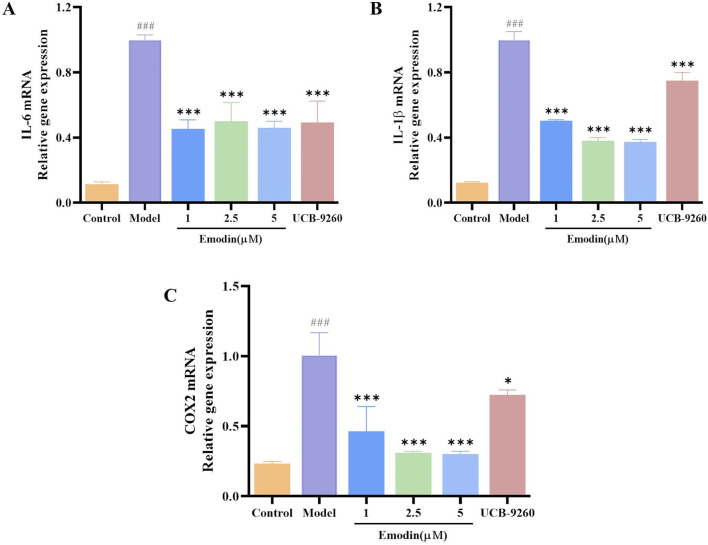
Emodin suppresses *IL-6*, *IL-1β*, and *COX2* mRNA levels in TNF-α-induced MH7A cells. **(A)** IL-6 levels in MH7A cells. **(B)** IL-1β levels in MH7A cells. **(C)** COX2 levels in MH7A cells. ^###^ *p <* 0.001 compared to the control group; *p < 0.05, ***p < 0.001 compared to the model group.

We have discovered that emodin attenuates the activation of the NF-κB signaling pathway by interacting with TNF-α. To evaluate whether emodin could suppress downstream inflammatory markers, RT-qPCR was employed to measure the expression of *IL-6*, *IL-1β*, and *COX2* mRNA levels in TNF-α-induced MH7A cells. Results revealed that emodin significantly reduced the mRNA levels of *IL-6*, *IL-1β*, and *COX2* compared to the model group (*p* < 0.001, Figures 10A–C). These findings suggest that emodin effectively suppresses the NF-κB-induced inflammatory response by interacting with TNF-α.

## 4 Discussion

Emodin has significant clinical application value in various diseases, such as RA, pancreatitis, and glomerulonephritis (Shrimali et al., 2013). However, it was unclear how it exerts anti-RA effects. This study first confirmed the anti-RA effect of emodin using the CIA rat model, and then employed RNA sequencing and thermal proteome profiling to elucidate the underlying mechanisms. RNA-seq analysis identified 83 differentially expressed genes, and KEGG pathway analysis of these DEGs indicated significant enrichment of the TNF signaling pathway. Additionally, TPP analysis revealed alterations in the abundance of 37 proteins following emodin treatment, with TNF-α exhibiting the most notable change. Subsequent KEGG pathway analysis also confirmed the enrichment of the TNF signaling pathway. These results suggest that the TNF signaling pathway, particularly TNF-α, may be critical targets and pathways through which emodin exerts its anti-RA effects.

TNF-α can activate the immune system and maintain normal immune responses in the body (Horiuchi et al., 2010). Dysregulated or excessive secretion of TNF-α is implicated in a variety of diseases, including RA as highlighted by multiple studies (Gandhi et al., 2021; Liao et al., 2023). Moreover, TNF-α has been identified as a critical target in RA, influencing inflammation through mechanisms involving pathways such as NF-κB (Jimi et al., 2019). Consequently, by integrating RNA-seq and TPP data, we aim to explore the anti-RA mechanisms of emodin from the perspective of TNF-α inhibition.

L929 cells are particularly susceptible to necroptosis when stimulated by TNF-α, making them an ideal model for identifying compounds that can inhibit TNF-α function (Sun et al., 2020; Dietrich et al., 2021). In this study, emodin significantly enhanced the survival rate and cell viability of TNF-α-induced L929 cells, suggesting that emodin may interact with TNF-α. To explore the possibility that emodin might exert its effects by promoting cell survival, the following experiments were conducted as described previously (Ma et al., 2014). First, we evaluated the impact of various concentrations of emodin on L929 cell viability. The results showed that emodin did not promote L929 cell growth at concentrations below 15 μM. Next, we performed an cytotoxicity assay using Dox (Doxorubicin) and ActD to induce cell damage in L929 cells and observed that emodin did not mitigate the cytotoxic effects of these agents. These results suggest that emodin does not enhance the viability of TNF-α-induced L929 cells through mechanisms such as promoting cell growth or inhibiting apoptosis (Supplementary Figures S4, S5).

To investigate the binding interaction between emodin and TNF-α, we utilized the DARTS and CETSA techniques. These methods are based on the principle that small molecules can enhance the thermal stability and protease resistance of their target proteins upon binding (Wu et al., 2021). Emodin significantly increased both the enzymatic and thermal stability of TNF-α, suggesting a strong binding interaction. To further confirm this interaction, AUF-LC/MS、SPR and BLI were employed. AUF-LC/MS, effective in screening compounds through their interaction with target proteins (Yang et al., 2022; Lan et al., 2021), identified emodin as a TNF-α-binding molecule. Moreover, SPR and BLI analysis, recognized by the United States Pharmacopeia as a definitive method for analyzing molecular interactions (Lv et al., 2022; United States Pharmacopeia, 2021), revealed a KD of 26.49 μM and 26.67 μM for the emodin-TNF-α interaction, confirming its strength and specificity. Collectively, these findings corroborate the robust interaction between emodin and TNF-α.

TNFR1 is widely expressed with a conserved death domain motif (Aggarwal et al., 2012). TNF-α binds to TNFR1 in symmetric trimeric form, activating pathways such as NF-κB signaling which lead to inflammation (Tang et al., 1996). However, in its asymmetric trimeric form, TNF-α can only bind to two TNFR1 receptors, preventing full signal transduction (McMillan et al., 2021). This study found that the hydrogen bonding interactions between emodin and 6ooy amino acids LEU-57, TYR-119, and ELY-122 enhance the stability of the asymmetric trimer of TNF-α, as indicated by lower average RMSD, Rg, SASA, and RMSF values. Consequently, this stabilization prevents TNF-α from effectively interacting with TNFR1, a finding further supported by ELISA and BLI results.

The disruption of TNF-α-TNFR1 interaction blocks NF-κB activation. NF-κB is a transcription factor activated by TNF-α (Mitchell et al., 2016). Typically, NF-κB remains inactive in the cytoplasm, bound to IκBα, and is activated through phosphorylation and subsequent degradation of IκBα, facilitating the translocation of p65 subunits to the nucleus (Campbell et al., 2013). Additionally, previous studies have shown that NF-κB activation in the synovial tissue of RA patients triggers inflammatory responses, leading to increased production of cytokines such as TNF-α, IL-6, IL-1β, and COX2, which amplify the inflammatory response (Kondo et al., 2021). Given that emodin effectively binds to TNF-α, thereby antagonizing its activity, we investigated its potential to inhibit TNF-α-induced NF-κB activation. Using a luciferase reporter gene assay, we first assessed the impact of emodin on the NF-κB signaling pathway. Subsequently, the classic TNF-α-induced MH7A cell model, commonly used to study anti-RA effects, was employed to corroborate these findings. Our study found that emodin inhibited NF-κB reporter gene expression in TNF-α-induced HEK293T cells, decreased the phosphorylation of p65 and IκBα as well as the nuclear translocation of p65 and reduced the transcriptions of IL-6, IL-1β, and COX2 in TNF-α-induced MH7A cells. These results suggest that emodin suppresses the NF-κB signaling pathway through its effects on TNF-α, thereby reducing the expression of inflammatory mediators and exerting anti-RA effects.

Under normal physiological conditions, the production and release of TNF-α are tightly regulated (Brenner et al., 2015). However, in response to both external and internal stimuli, TNF-α secretion increases. The symmetric trimer of TNF-α binds to TNFR1, triggering downstream signaling pathways such as NF-κB, MAPK, and apoptotic signaling, which leads to a cascade of events, including the release of inflammatory cytokines, tissue degeneration, and host defense (Jang et al., 2021). In contrast, the asymmetric trimer of TNF-α lacks these activities. Our research found that emodin can directly bind to TNF-α, stabilizing its asymmetric trimer and inhibiting the interaction between TNF-α and TNFR1. This inhibition prevents the phosphorylation and nuclear translocation of p65, and subsequently suppresses the upregulation of inflammatory cytokines such as IL-6 and IL-1β, thereby exerting an anti-RA effect. However, whether emodin also modulates other related pathways, such as MAPK and apoptotic signaling, to exert its anti-RA effect warrants further investigation.

Consequently, it can be inferred that emodin’s inhibitory effect on TNF-α activity is primarily due to its stabilization of the TNF-α asymmetric trimer, which potentially disrupts the full activation of downstream inflammatory signaling pathways, presenting a novel approach to managing RA distinct from traditional therapies that broadly neutralize TNF-α activity.

## 5 Conclusion

Our study conclusively demonstrates that emodin effectively targets and modulates the activity of TNF-α, a pivotal inflammatory mediator in RA. By binding directly to TNF-α and stabilizing its asymmetric trimeric form, emodin inhibits the subsequent activation of the NF-κB signaling pathway. This specific interaction leads to a marked reduction in the expression of key inflammatory cytokines. These molecular insights are corroborated by our *in vivo* findings, where emodin significantly alleviated symptoms in a collagen-induced arthritis rat model, highlighting its potent anti-inflammatory and anti-arthritic effects. Our findings provide a robust theoretical foundation for the further development and clinical evaluation of emodin as a promising anti-RA agent.

## Data Availability

The datasets presented in this study can be found in online repositories. The names of the repository/repositories and accession number(s) can be found in the article/Supplementary Material.

## Glossary

RA: rheumatoid arthritis
CIA: collagen-induced arthritis
ELISA: enzyme-linked immunosorbent assay
RF: rheumatoid factor
TNF-α: tumor necrosis factor-alpha
IL-6: interleukin-6
IL-1β: interleukin-1β
IL-8: interleukin-8
HE: Hematoxylin and eosin
TPP: thermal proteome profiling
BLI: bio-layer interferometry
DARTS: drug affinity responsive target stability
CETSA: cellular thermal shift assay
AUF-LC/MS: affinity ultrafiltration-liquid chromatography/mass spectrometry
SPR: surface plasmon resonance
RT-qPCR: real-time quantitative polymerase chain reaction
COX2: cyclooxygenase 2
p-p65: phosphor-p65
p-IκBα: phospho-IκBα
NF-κB: nuclear factor-kappa B
FLS: fibroblast-like synoviocyte
MAPK: mitogen-activated protein kinase
*P. forrestii*: *Periploca forrestii*
LPS: lipopolysaccharide
MTX: methotrexate
IFA: incomplete Freund’s adjuvant
Act D: Actinomycin D
CMC-Na: carboxymethyl cellulose
FBS: fetal bovine serum
RMSD: root mean square deviation
Rg: radius of gyration
SASA: solvent accessible surface area
RMSF: root mean square fluctuation
LTβR: lymphotoxin β receptor
RANK: receptor activator of NF-κB
mTNF-α: membrane-bound TNF-α
sTNF-α: soluble TNF-α
TNFR1: tumor necrosis factor-α receptor 1
TNFR2: tumor necrosis factor-α receptor 2
SA: streptavidin
HRP: horseradish peroxidase
Dox: doxorubicin
