# Supplementary material for: Emodin mitigates rheumatoid arthritis through direct binding to TNF-α
Source: Front Pharmacol. 2025 Feb 26;16:1520281. doi: 10.3389/fphar.2025.1520281 (PMC11896996; doi:10.3389/fphar.2025.1520281)
Supplement: Supplementary file 1 [file DataSheet1.docx]

Supplementary Material

# 1. Supplementary Data

The data supporting this article are available within the article itself and in its supplementary materials. Additionally, the transcriptomic data have been deposited in the Sequence Read Archive database (PRJNA1106217).

1. **Supplementary Figures**

| 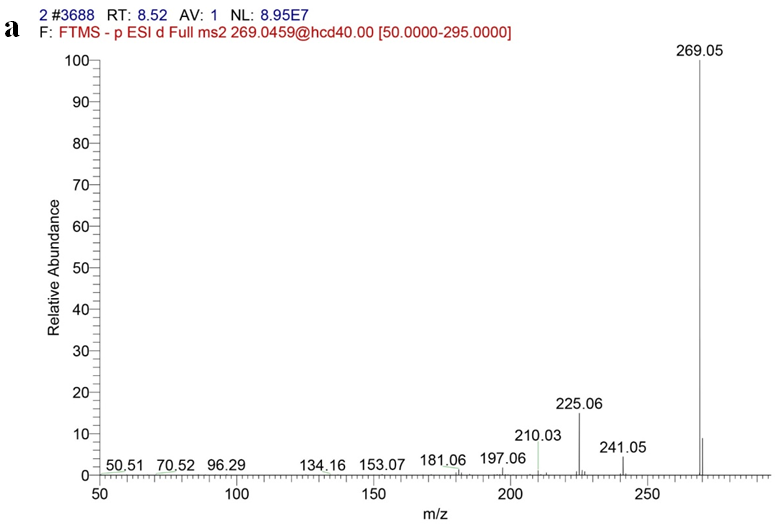 | 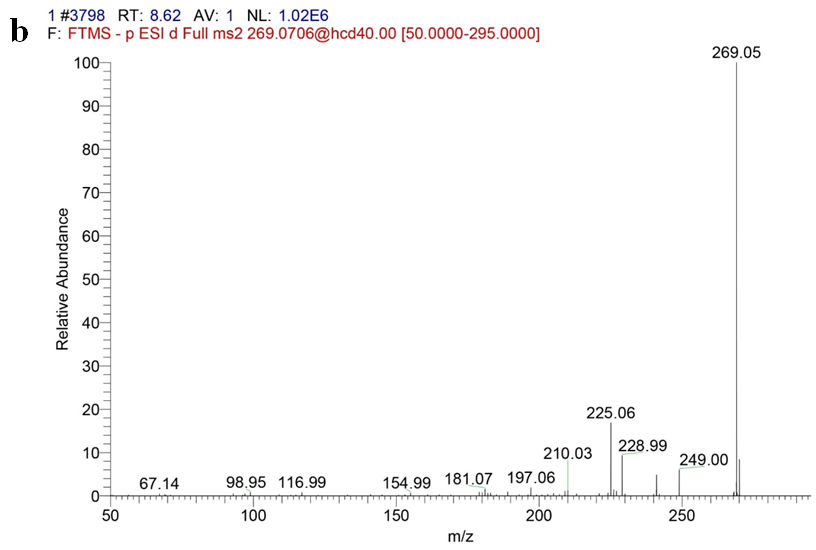 |
| --- | --- |
| 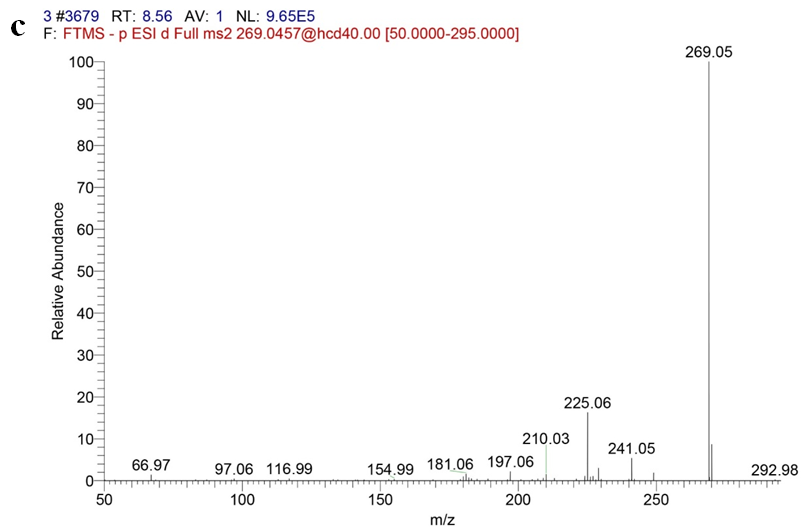 | 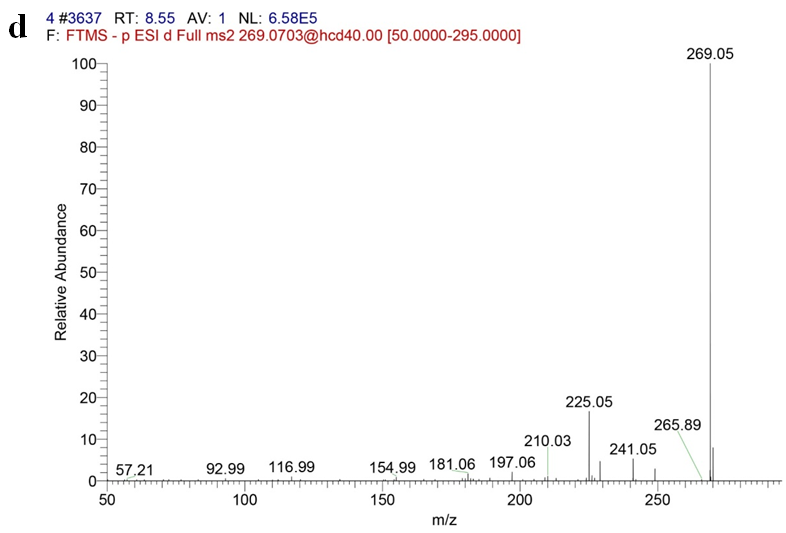 |

**Figure S1**. Secondary mass spectrum. (a) standard substance, (b) *P. forrestii* extract group, (c) TNF-α/*P. forrestii* group, (d) Control group.

A

|  | |  | Repeat 1 | | | | | | |  | Repeat 2 | | | | | | |  | Repeat 3 | | | | | | |
| --- | --- | --- | --- | --- | --- | --- | --- | --- | --- | --- | --- | --- | --- | --- | --- | --- | --- | --- | --- | --- | --- | --- | --- | --- | --- |
| Emodin | TNF-α  26 kDa | 130  95  72  55  43  34  26  17 |  | | | | | | | 130  95  72  55  43  34  26  17 | 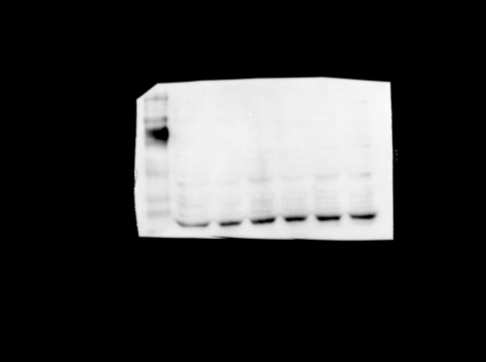 | | | | | | | 130  95  72  55  43  34  26  17 |  | | | | | | |
| DMSO | TNF-α  26 kDa | 130  95  72  55  43  34  26 |  | | | | | | | 130  95  72  55  43  34  26  17 | 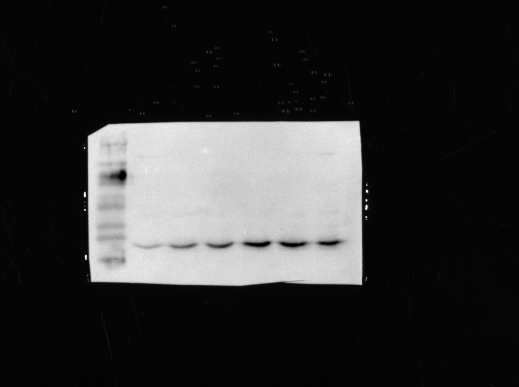 | | | | | | | 130  95  72  55  43  34  26  17 |  | | | | | | |
|  | |  |  | undiluted | 1:100 | 1:300 | 1:1000 | 1:3000 | 1:10000 |  |  | undiluted | 1:100 | 1:300 | 1:1000 | 1:3000 | 1:10000 |  |  | undiluted | 1:100 | 1:300 | 1:1000 | 1:3000 | 1:10000 |

**B**

|  | |  | Repeat 1 | | | | | | | |  | Repeat 2 | | | | | | | |  | Repeat 3 | | | | | | | |
| --- | --- | --- | --- | --- | --- | --- | --- | --- | --- | --- | --- | --- | --- | --- | --- | --- | --- | --- | --- | --- | --- | --- | --- | --- | --- | --- | --- | --- |
| Emodin | TNF-α  26 kDa | 180  130  95  72  55  43  34  26  17  10 | 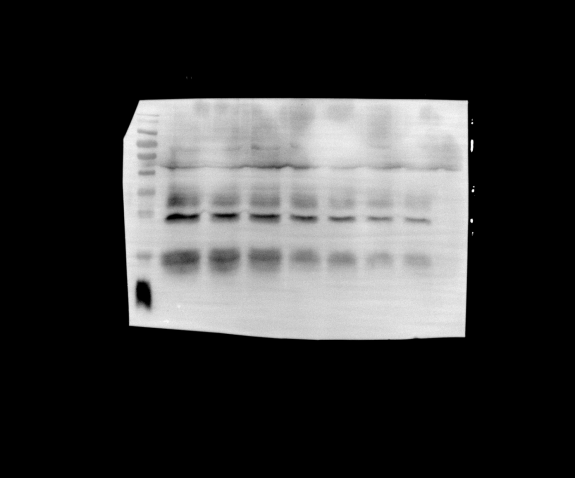 | | | | | | | | 130  95  72  55  43  34  26  17  10 | 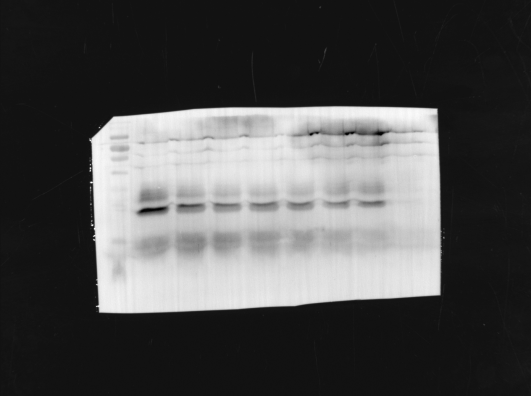 | | | | | | | | 180  130  95  72  55  43  34  26  17  10 | 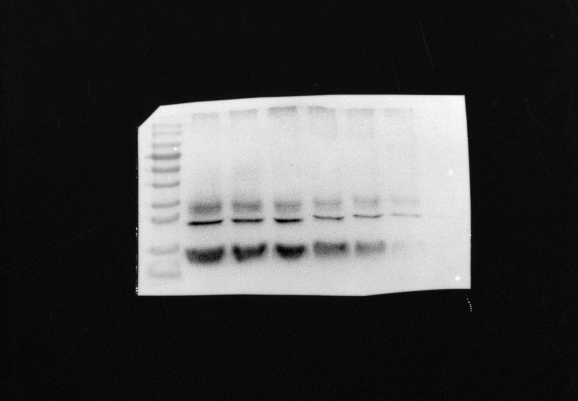 | | | | | | | |
| DMSO | TNF-α  26 kDa | 180  130  95  72  55  43  34  26  17  10 | 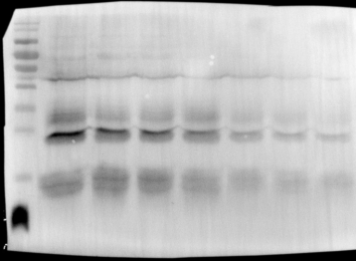 | | | | | | | | 95  72  55  43  34  26  17  10 | 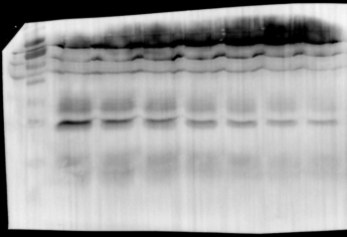 | | | | | | | | 95  72  55  43  34  26  17  10 |  | | | | | | | |
|  | |  |  | RT | 60 ℃ | 64 ℃ | 68 ℃ | 72 ℃ | 76 ℃ | 80 ℃ |  |  | RT | 60 ℃ | 64 ℃ | 68 ℃ | 72 ℃ | 76 ℃ | 80 ℃ |  |  | RT | 60 ℃ | 64 ℃ | 68 ℃ | 72 ℃ | 76 ℃ | 80 ℃ |

**Figure S2**. Example of original western blot for three repeats. Images of full blots of the samples shown in Figure 5.

|  |  | Repeat 1 | | | | | | |  | Repeat 2 | | | | | | | |  | Repeat 3 | | | | | | | |
| --- | --- | --- | --- | --- | --- | --- | --- | --- | --- | --- | --- | --- | --- | --- | --- | --- | --- | --- | --- | --- | --- | --- | --- | --- | --- | --- |
| GAPDH 36 kDa | 48  35  25 |  | | | | | | |  |  | | | | | | | | 48 | 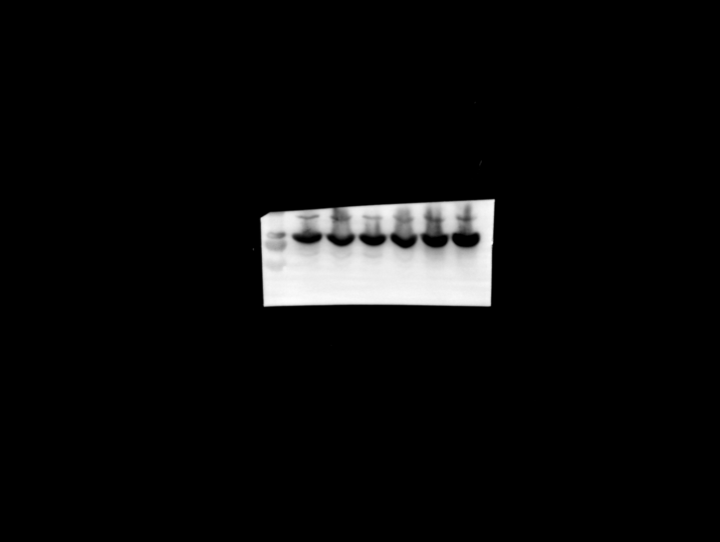 | | | | | | | |
|  |  |  |  |  |  |  |  |  | 48  35  25 |  |  |  |  |  |  |  |  | 35  25 |  |  |  |  |  |  |  |  |
|  |  |  |  |  |  |  |  |  |  |  |  |  |  |  |  |  |  | 17 |  |  |  |  |  |  |  |  |
|  |  |  |  |  |  |  |  |  |  |  |  |  |  |  |  |  |  |  |  |  |  |  |  |  |  |  |
| P-IkB 39 kDa |  |  | | | | | | |  | 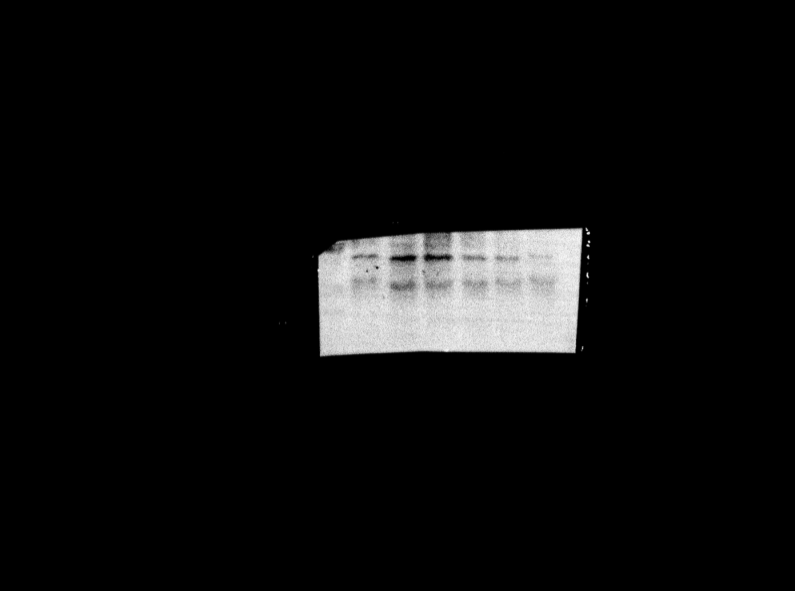 | | | | | | | | 48  35  25 | 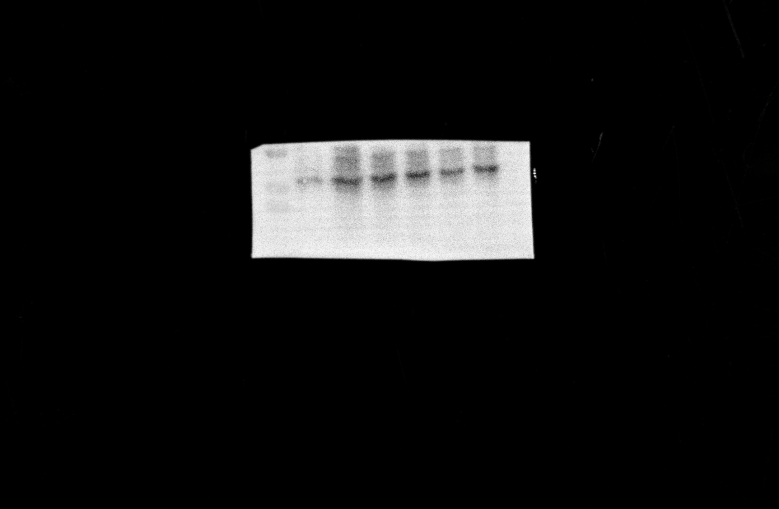 | | | | | | | |
|  | 48  35  25  17 |  |  |  |  |  |  |  | 48  35  25 |  |  |  |  |  |  |  |  |  |  |  |  |  |  |  |  |  |
|  |  |  |  |  |  |  |  |  |  |  |  |  |  |  |  |  |  |  |  |  |  |  |  |  |  |  |
| IkB 40 kDa |  |  | | | | | | |  | 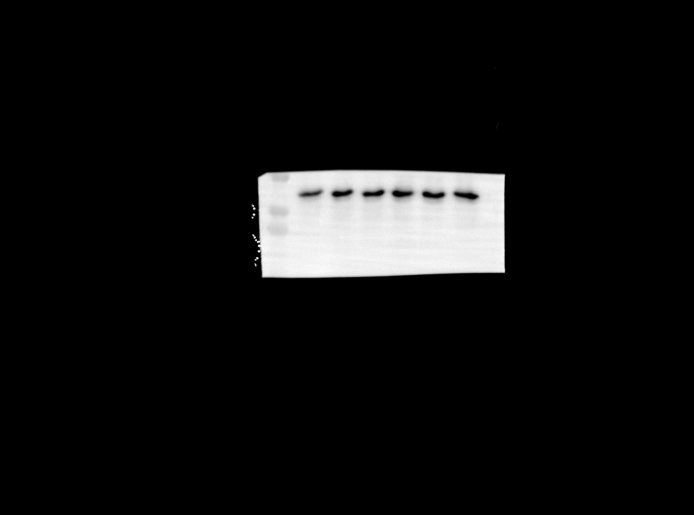 | | | | | | | |  | 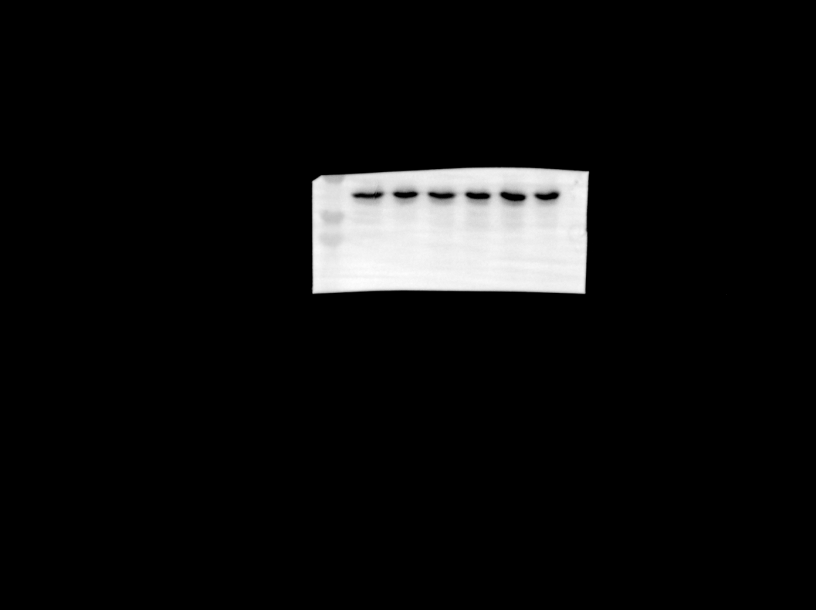 | | | | | | | |
|  | 48  35  25  17 |  |  |  |  |  |  |  | 48  35  25  17 |  |  |  |  |  |  |  |  | 48  35  25  17 |  |  |  |  |  |  |  |  |
|  |  |  |  |  |  |  |  |  |  |  |  |  |  |  |  |  |  |  |  |  |  |  |  |  |  |  |
| p-p65 65 kDa | 135  100  75  63 |  | | | | | | | 135  100  75  63 |  | | | | | | | | 180  135  100  75  63 |  | | | | | | | |
| P65 65 kDa | 135  100  75  63 |  | | | | | | | 135  100  75  63 | 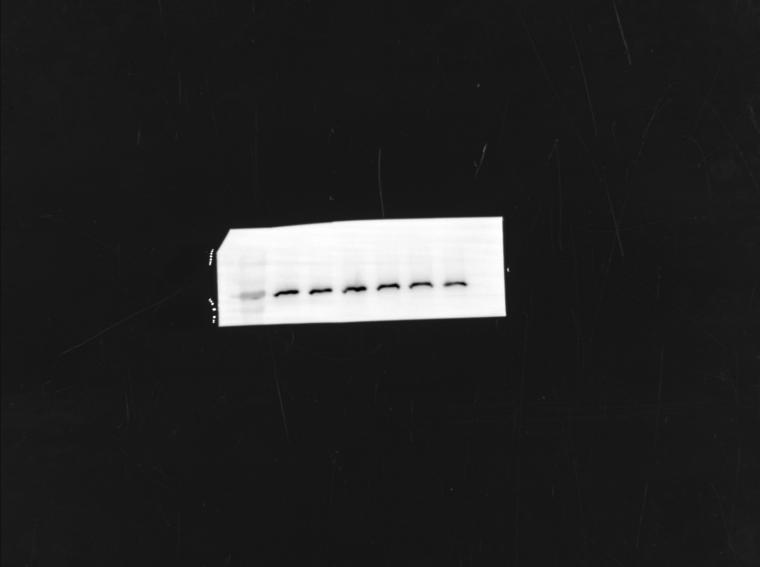 | | | | | | | | 100  75  63  48 | 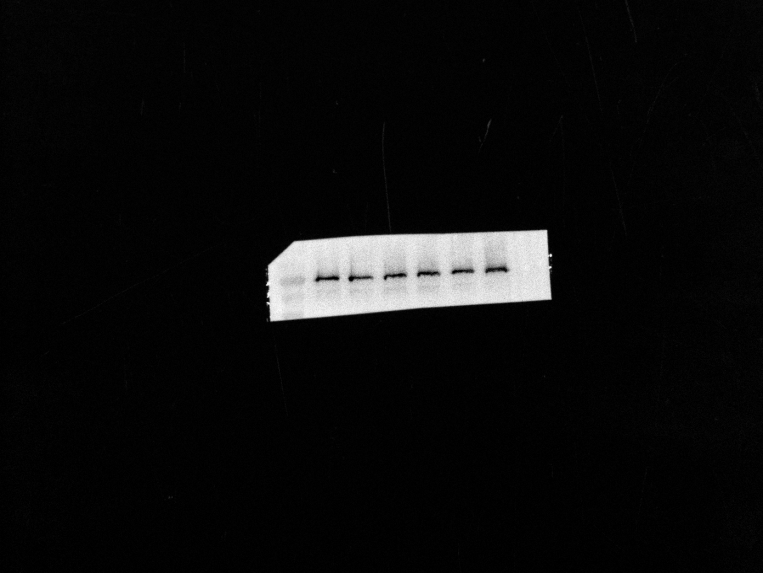 | | | | | | | |
|  |  |  | Control | Model | Emodin 1 μM | Emodin 2.5 μM | Emodin 5 μM | UCB-926 |  |  | Control | Model | Emodin 1 μM | Emodin 2.5 μM | Emodin 5 μM | UCB-926 |  |  |  | Control | Model | Emodin 1 μM | Emodin 2.5 μM | Emodin 5 μM | UCB-926 |  |

**Figure S3**. Example of original western blot for three repeats. Images of full blots of the samples shown as cropped images in Figure 10. Of note, in some instances the membranes were cut prior staining in order to ensure simultaneous staining with more than one antibody against proteins of different molecular weights.


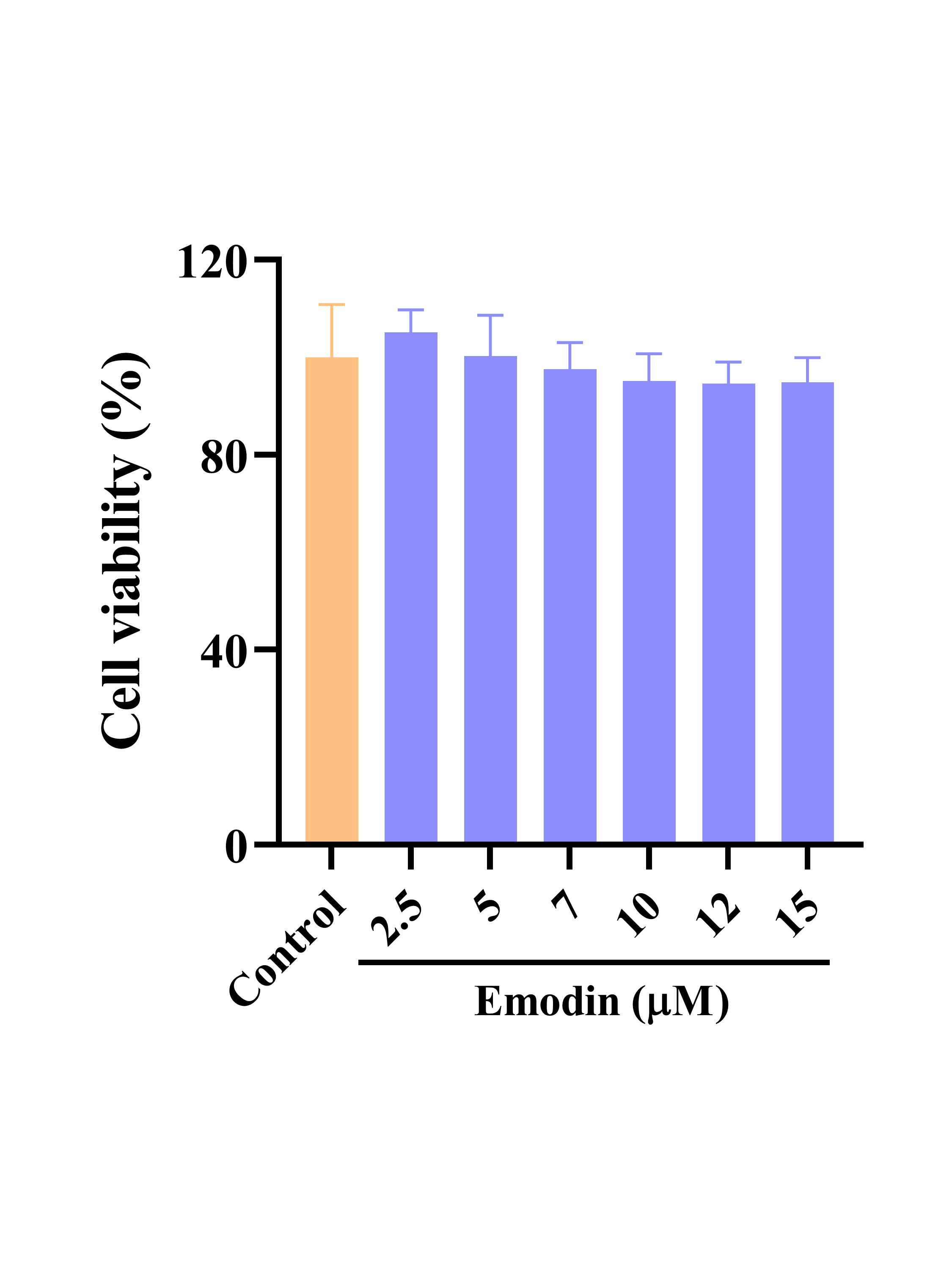


**Figure S4. Effect of emodin on the L929 cell viability.**


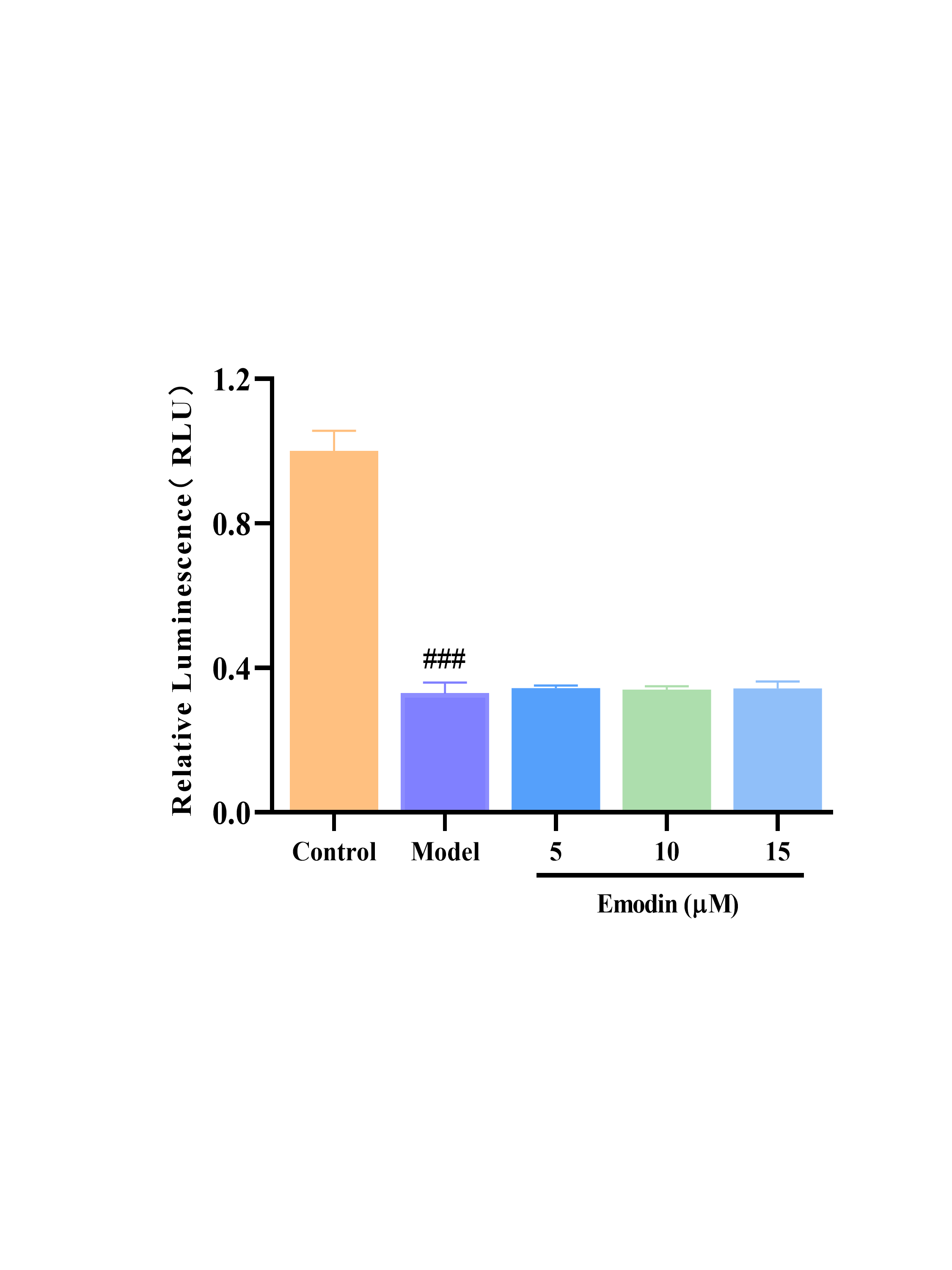


**Figure S5. Emodin’s effect on the viability of DOX-treated L929 cells**. ^###^*p* < 0.001 compared to the control group

**
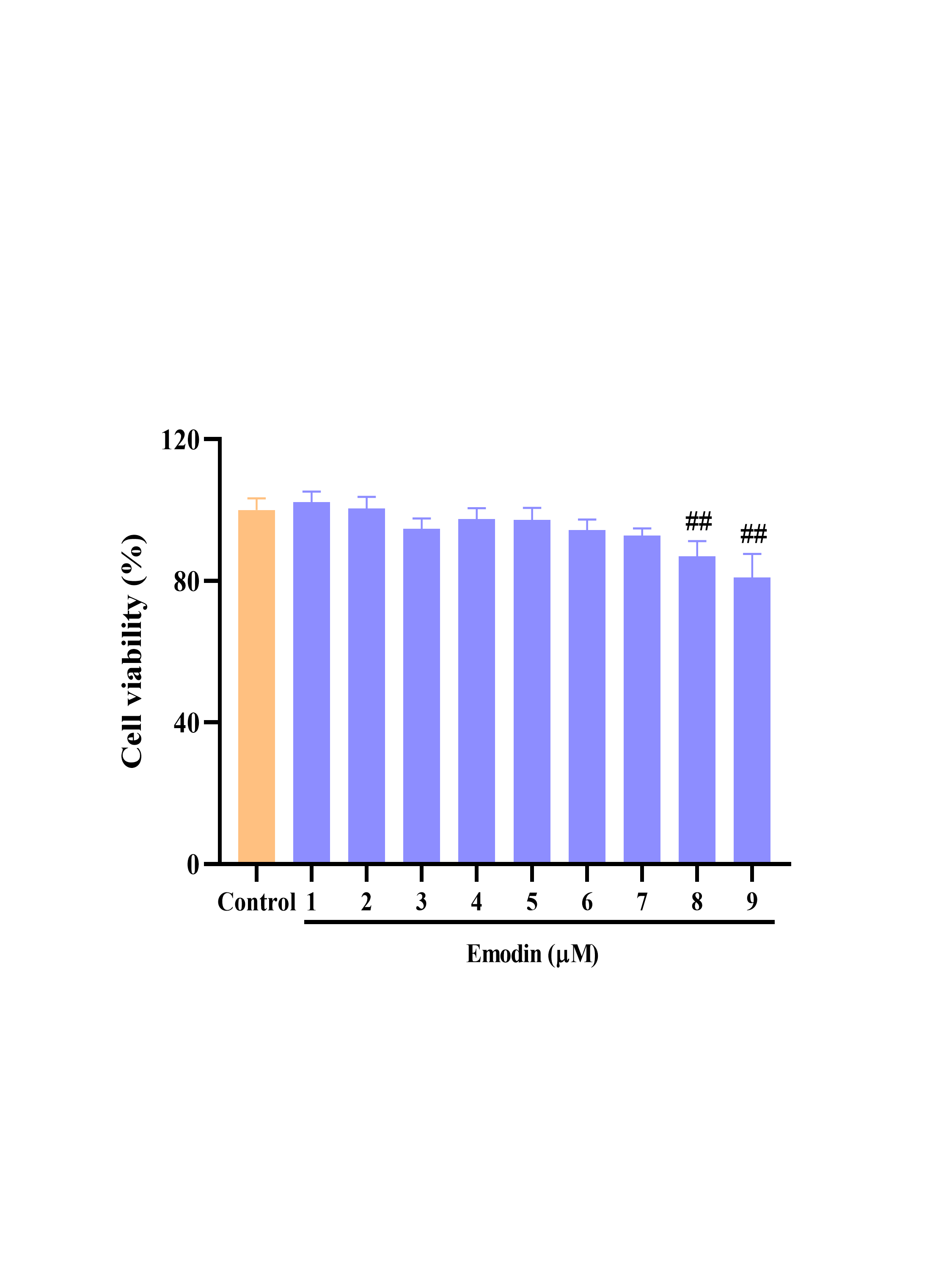
**

**Figure S6. Effect of emodin on the MH7A cell viability**. ^##^*p* < 0.01 compared to the control group


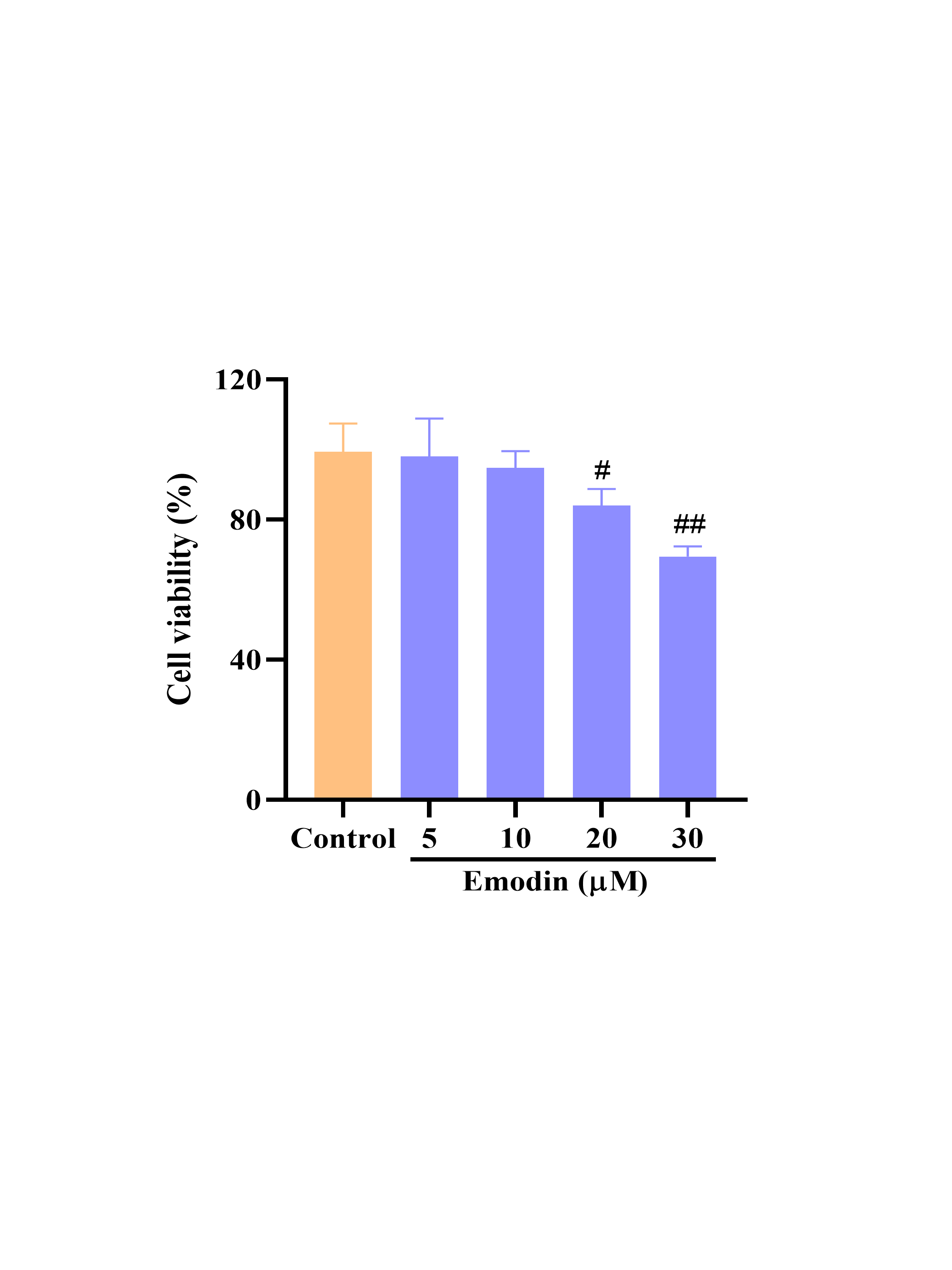


**Figure S7. Effect of emodin on the HEK293T cell viability**. ^#^*p* < 0.05, ^##^*p* < 0.01 compared to the control group
